# Theoretical analysis of neuronal network’s response under different stimulus

**DOI:** 10.1371/journal.pone.0314962

**Published:** 2024-12-20

**Authors:** Haosen Xue, Zeying Lu, Yueheng Lan, Lili Gui, Xiaojuan Sun

**Affiliations:** 1 School of Science, Beijing University of Posts and Telecommunications, Beijing, China; 2 School of Electronic Engineering, Beijing University of Posts and Telecommunications, Beijing, China; 3 Key Laboratory of Mathematics and Information Networks(Beijing University of Posts and Telecommunications), Ministry of Education, Beijing, China; Lanzhou University of Technology, CHINA

## Abstract

Neuromodulation plays a critical role in the normal physiological functions of organisms. With advancements in science and technology, neuromodulation has expanded into various fields. For instance, in the field of engineering, in vitro-cultured neural networks are utilized to perform closed-loop control for achieving complex functionalities. Conducting pioneering theoretical research using mathematical models is particularly essential for enhancing efficiency and reducing costs. This study focuses on examining the relationship between input and output in order to establish a groundwork for more advanced closed-loop regulation applications in engineering. Using a constructed neural network model, Poisson, square wave and direct current (DC) stimulation are applied. The results show that the network’s firing rate increases with the frequency or amplitude of these stimulations. And the network’s firing rate could reach to a stable state after the stimulation is applied for 0.8s and return to initial states when the stimulus is removed for 1s. To ascertain if the system exhibits a memory effect from the previous stimulus, we conduct independent and continuous stimulation schemes. Comparing the firing rate of neuronal networks under these two stimulation schemes reveals a memory effect of the system on the previous stimulus, which is independent of network properties and stimulus types. Finally, by applying square wave stimulation to the in vitro cultured neural network, we have confirmed that cultured neural network actually can reach to a steady state and have memory effects on the previous stimulus. Our research results have important theoretical significance and reference value for designing the closed-loop regulation strategy of in vitro cultured neuronal networks.

## Introduction

External stimuli can trigger physiological and biochemical responses in organisms through specific signal transduction pathways, which regulate the function and behavior of cells, tissues, or the entire organism. Stimulus modulation is an integral part of living organisms’ adaptive response to the external environment, aiming to maintain homeostasis and promote survival. Stimulus modulation can extend from the human body to the field of engineering. Currently, many engineering applications utilize in vitro-cultured neural networks and organoids for closed-loop modulation, referring to neuromodulation in the human body. This allows the application of neuromodulation in engineering to achieve complex functions, such as speech recognition and robotic arm control, for various practical applications [1–4]. The basis of closed-loop regulation is to comprehend the input-output relationship. However, the use of in vitro culture in engineering for experimental investigations is time-consuming, incurs relatively high capital costs, and poses challenges in accurately measuring experimental data [5–7]. Therefore, the use of simulation for preliminary investigations is crucial, saving time and economic costs while easily regulating the numerous parameters needed for repetitive simulations, and ensuring security. Effective execution of closed-loop control requires the discussion of the dynamic behavior of neural networks, with the input-output relationship as its foundation. In the past research, many scholars have devoted themselves to exploring the input-output laws and stimulus regulation mechanisms of neuronal networks.

At the turn of the 21st century, numerous research teams both domestically and internationally delved into the realm of neuromodulation. Utilizing real neural networks cultured in vitro, they delved into the impact of both pressor and endogenous signals on the relationship between input and output [8, 9]. By observing the correlation between the firing spike trains of two neurons with correlated inputs, they unveiled the intricate dynamics between output correlation and input correlation [10]. Furthermore, they delved into modulating the properties of corticospinal pathways through the shape of the input-output relationship [11]. Additionally, they harnessed live neurons to steer simulated animals, mobile robots, and drawing arms, thereby advancing artificial intelligence [1]. By employing rat neocortical cells alongside various stimulation protocols, they uncovered the efficacy of distributed stimulation in modulating cell bursting in vitro, offering promising avenues for epilepsy treatment [12]. Moreover, they achieved the feat of imparting learning and flight control capabilities to neural networks through external stimulation [13].

In recent years, researchers have further delved into the realm of neuromodulation. Through the application of a method measuring the covariation of excitatory and inhibitory inputs received by the cell, studies have demonstrated that input correlation noise indeed affects the accuracy of the response [14]. Additionally, the implementation of ‘light-clamp’ feedback control techniques, utilizing optical stimulation for precise and dynamic closed-loop neuronal control, has enabled the regulation of neuronal firing through feedback mechanisms [15]. Investigations into the relationship between input correlation and output correlation have involved manipulating the active and passive properties of dendrites [16]. Notably, Brett J. Kagan’s team developed the DishBrain system, harnessing the intrinsic adaptive computational capabilities of neurons in a structured environment to emulate the behavior of humans playing ping-pong. This system demonstrated that an ex vivo cortical neuronal layer can self-organize its activities to exhibit intelligent behavior in a simulated game world [2, 17]. Furthermore, Yuichiro Yada’s team constructed a closed-loop experimental setup for physical savings pool computation of live neuron cultures, enabling vehicular robots to solve mazes based on coherent signal outputs from these cultures [3]. Takuma Sumi’s team utilized primary rat cortical neurons to create a micropatterned biological neural network (mBNN). Through mBNN-based savings pool computation, they achieved classification of static spatial patterns, spatio-temporal signals, and even human speech digits, with the modular structure of the mBNN enhancing classification accuracy [4]. In another advancement, Hongwei Cai’s team cultivated organoids from human stem cells and connected them to a high-density electrode array to construct the Brainoware system. This system can undergo unsupervised learning and be trained for speech recognition [18]. Most recent research predominantly centers on exploring complex engineering functionalities by utilizing real neural networks cultured in vitro. Given the imperative of comprehending neural network input-output relationships in advance to achieve specific functionalities, simulation serves as a preliminary tool for investigating these relationships. Consequently, this facilitates more refined closed-loop control design.

Previous studies on the relationship between input and output in neural networks have typically focused on specific influencing factors [8, 9, 14, 16]. In contrast, our research explores the effects of different types of stimuli, varying their frequencies, amplitudes, and intensities to comprehensively investigate the input-output relationship. Additionally, earlier studies have not considered whether neural networks retain memory of previous stimuli, which could impact the results [2, 12, 17]. Our research addresses this by incorporating both continuous and independent stimulation schemes to examine memory effects, thereby minimizing the potential influence of such effects on our findings. Moreover, prior research has often relied solely on either simulations or experiments [8–10, 12–16]. In contrast, our study integrates both approaches, enhancing the reliability and persuasiveness of our results.

In our current study, we explore the modulation of neural systems through both modeling simulation and real experiments, providing pioneering research for their application in closed-loop control in engineering. Through simplified neural network models, we investigate the activity changes of neurons under continuous input stimulation, accounting for the effects of spike-timing-dependent plasticity (STDP). Using the average firing rate of the neural network as a metric. By introducing various types of stimulation and manipulating the frequencies, amplitudes, and intensities of the stimulation, we observe an increase in the neural network’s firing rate with higher stimulation frequencies, amplitudes, or intensities. Due to the presence of synaptic plasticity mechanisms, it is necessary to consider whether the neural network exhibits memory of the stimulation and the time it takes for the neural network to reach stability after receiving or removing the stimulation. Through addressing these factors, we aim to provide theoretical groundwork for further real experiments in engineering, aiming to achieve precise closed-loop regulation of complex functionalities using in vitro cultured neural networks. Subsequently, we investigate the memory effects of the neural network. Measuring the time required for the neural network to stabilize after the introduction or removal of stimulation, we design two distinct stimulation schemes: independent and continuous. Comparing the differences in firing rates between the two stimulation schemes, we reveal memory effects within the neural network. Furthermore, through modifications in neural network properties and stimulation types, we further confirm the existence of memory effects and find that they are more pronounced under high-intensity or high-frequency stimulation. In order to mutually validate our simulation results, we also conduct experimental studies. By extensively researching the response characteristics of biological neural networks to stimulation using cultured real neural networks composed of neurons, we find that the firing rate of real neural networks increases with higher stimulation frequencies, consistent with the conclusions drawn from simulations. Similarly, in the exploration of memory effects, we observe corresponding conclusions, indicating the existence of memory effects in real neural networks, with more pronounced effects observed under high-frequency stimulation. This paper primarily adopts a combined approach of simulation and experimentation, thereby enhancing the persuasiveness of our research findings.

These studies not only contribute to a better understanding of the function and regulatory mechanisms of the nervous system, but also provide important pioneering studies for the application of closed-loop modulation in engineering. Through the combination of theory and experiment, we can more accurately grasp the input-output relationship of the nervous system, and thus achieve precise stimulus modulation strategies, which can play an important role in more fields such as drug screening, disease treatment and brain-computer interfaces, etc. These efforts will hopefully drive even more important breakthroughs in the fields of neuroscience and neural engineering in the future.

## Materials and methods

To explore the relationship between input and output, we establish an E-I random network model in this study. The network consists of 100 neurons, with an excitatory to inhibitory neuron ratio of 4:1. The initial connection probabilities for E-E, E-I, I-E, and I-I synapses are set to 0.3, 0.9, 0.9, and 0.9, respectively [19–22]. Each neuron is modeled using the adaptive exponential integrate-and-fire(AdEx) model to accurately describe the dynamic behavior of membrane potentials under different inputs. Synaptic plasticity between neurons is modeled using STDP, making the neural network model more biologically realistic. Three types of stimuli—Poisson, square wave, and direct current are applied to the network as inputs, with the average firing rate of the neural network chosen as the output. This choice aligns with biological rationale, as it reflects the degree of neuron activation.

### Neuron model

The neuronal model is AdEx model. The model parameters are carefully selected based on existing literature to ensure biological plausibility and consistency with previous research findings [23].

$$\begin{array}{cc} & C\frac{dv}{dt}=-g_{L}(v-E_{L})+g_{L}\Delta t\;\text{exp}\;(\frac{v-V_{t}}{\Delta t})-z+h \end{array}$$ (1)

$$\begin{array}{cc} & \tau_{z}\frac{dz}{dt}=a(v-E_{L})-z \end{array}$$ (2)

where *h* is the input current of the neuron, *z* is the adapting current, *g*_*L*_ is the leakage conductance, *E*_*L*_ is the leakage reversal potential, *V*_*s*_ is the neuron’s threshold potential, Δ*t* is the threshold slope factor, *a* is the adaptive coupling parameter, *τ*_*z*_ is the adaptive time constant and *C* is the membrane capacitance. When the membrane potential exceeds the threshold, i.e., *v* > *V*_*s*_, an action potential is generated, and then *v* and *z* are reset according to the following rule:

$$\begin{array}{c} v\leftarrow V_{r}\\ z\leftarrow z+b \end{array}$$

where *V*_*r*_ is the reset potential and *b* is the spike-triggered adaptive constant(See Table 1 for specific parameters).

**Table 1:** Neuronal parameter.

parameter	excitatory neuron	inhibitory neuron
C(*F*)	2.81 e-10	2.81 e-10
*g*_*L*_(*S*)	3 e-08	(3 ± 0.3) e-08
Δ*t*(*s*)	2 e-03	2 e-03
*E*_*L*_(*mV*)	-65	-65.6 ± 5
*V*_*r*_(*mV*)	-50.4	-50.4
a	4 e-09	(3.7028 ± 0.2) e-09
b	0.0805 e-09	0
*τ*_*z*_(*s*)	144 e-03	(149 ± 5) e-03
*V*_*s*_(*mV*)	-40.4	-40.4

*C*, the membrane capacitance;*g*_*L*_, the leakage conductance;Δ*t*, the threshold slope factor;*E*_*L*_, the leakage reversal potential;*V*_*r*_, the reset potential;a, the adaptive coupling parameter;b, the spike-triggered adaptive constant;*τ*_*z*_, the adaptive time constant;*V*_*s*_, the neuron’s threshold potential.

### Synaptic model

Neurons receive inputs from other neurons, the inputs are classified into excitatory and inhibitory inputs, excitatory synapses are based on AMPA receptors and inhibitory synapses are based on GABA receptors. Neurons receive three types of inputs, one is external and the other two types of inputs are recurrent inputs, from excitatory and inhibitory synapses. Only the recurrent inputs from excitatory neurons are considered, modelled as conductance-based forms. The synaptic model and parameters used in this study refer to previous literature, ensuring consistency with biological reality. This approach enhances the accuracy and reliability of our model [24–26].

#### Synaptic strength

The strength of the synaptic connection between neuron *i* and neuron *j* is denoted as $w_{ij}^{AB}$, where *K* ∈ {*exi*, *inh*}, *j* denotes the pre-synaptic neuron and *i* denotes the post-synaptic neuron. Synaptic strength is modelled as:

$$\begin{array}{c} w_{ij}^{AB}=\{\begin{array}{cc} \frac{J^{EE}W_{ij}}{N_{E}} & A=exi,B=exi\\ \frac{J^{EI}W_{ij}}{N_{I}} & A=exi,B=inh\\ \frac{J^{IE}W_{ij}}{N_{E}} & A=inh,B=exi\\ \frac{J^{II}W_{ij}}{N_{I}} & A=inh,B=inh \end{array} \end{array}$$ (3)

where *N*_*E*_ and *N*_*I*_ are the number of presynaptic neurons. In the simulation model *J*^*EE*^ = 1, *J*^*EI*^ = 1.5, *J*^*IE*^ = 3, *J*^*II*^ = 1.5 (in *nS*) and *W*_*ij*_ is a uniform distribution on [0.5,1].

#### Excitatory neuronal input

$$\begin{array}{c} h_{i}^{exi}=I_{i}^{ext}+I_{i}^{exi}+I_{i}^{inh} \end{array}$$ (4)

$$\begin{array}{c} I_{i}^{exi}=G_{AMPA}\cdot (-v+E_{AMPA})(\sum_{j}w_{ij}^{exi}g_{j}^{AMPA}\left(t\right)) \end{array}$$ (5)

$$\begin{array}{c} I_{i}^{inh}=G_{GABA}\cdot (-v+E_{GABA})(\sum_{j}w_{ij}^{inh}g_{j}^{GABA}\left(t\right)) \end{array}$$ (6)

$$\begin{array}{c} \frac{dg^{K}}{dt}=-\frac{g^{K}}{\tau_{K}}+F\cdot (1-g^{K})\sum_{f}\delta (t-t^{f}),K\in \left\{AMPA,GABA\right\} \end{array}$$ (7)

where *w*^{*exi*,*inh*}^ is the synaptic coupling strength, which varies with the synaptic plasticity STDP, $\sum_{j,f}\delta (t-t_{j}^{f})$ is the spike train, *g*^*K*^ ∈ [0, 1] is the relative conductance, *G*_*AMPA*_ and *G*_*AMPA*_ are scaling factors, and it should be noted that the time dynamics of the rising branch of the synaptic conductance is not considered here, only the time course of the decay, which subsequently decays with the time constant *τ*_*K*_. (see Table 2 for specific parameters).

**Table 2:** Synaptic parameters.

parameter	AMPA	GABA
G	20	20
E(mV)	0	-75
*τ*(ms)	4	10
F	0.5	0.5

G, scaling factors;e, reversal potential;*τ*, time constant;F, constant.

#### Inhibitory neuronal input

$$\begin{array}{c} h_{i}^{inh}=I_{i}^{ext}+I_{i}^{exi}+I_{i}^{inh} \end{array}$$ (8)

$$\begin{array}{c} I_{i}^{\text{exi}\;}=\sum_{j,f}w_{ij}^{exi}\delta (t-t_{j}^{f}) \end{array}$$ (9)

$$\begin{array}{c} I_{i}^{inh}=\sum_{j,f}w_{ij}^{inh}\delta (t-t_{j}^{f}) \end{array}$$ (10)

#### Synaptic plasticity

Synaptic plasticity in our model is implemented using STDP, a well-established biological mechanism that adjusts the strength of synapses based on the relative timing of spikes between pre- and post-synaptic neurons. Specifically, STDP strengthens synapses when a pre-synaptic neuron fires just before a post-synaptic neuron (long-term potentiation, LTP), and weakens them when the pre-synaptic neuron fires after the post-synaptic neuron (long-term depression, LTD). This timing-dependent adjustment is crucial for various learning and memory processes in biological neural systems.

**Fig 1:**
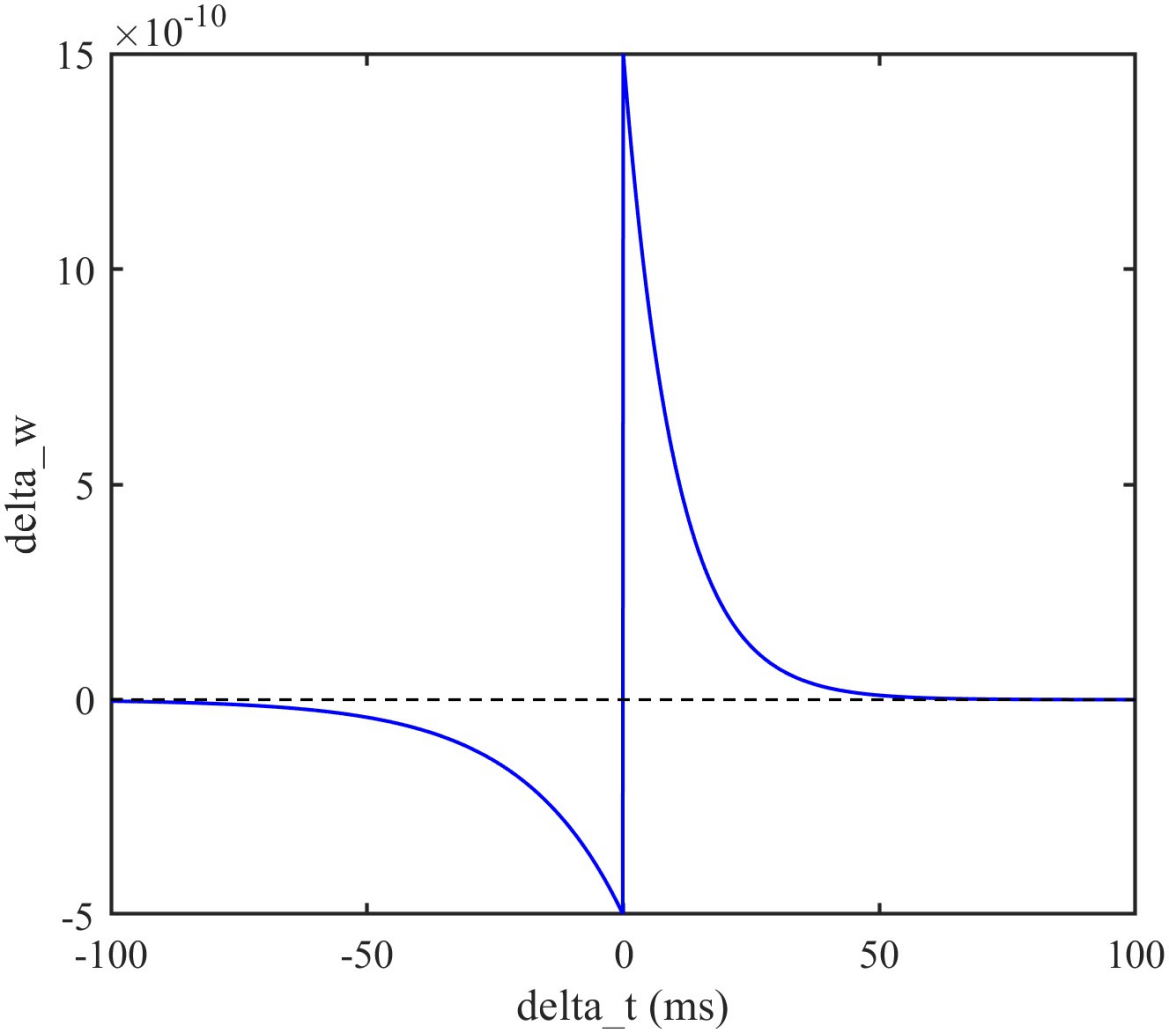
STDP time window. The relationship between the increment in synaptic strength and the difference in firing time. The values are *A* = 1, λ = 0.5, *u* = 0.3, *α* = 0.8, *τ*^+^ = 0.015, *τ*^*x*^ = 0.03, *τ*^−^ = 0.03, *w*_0_ is constant and depends on other parameters in the neural network.

In our study, we implemented the STDP model, which governs synaptic changes through an exponentially decaying function based on the time difference between pre- and post-synaptic spikes [27]. The model parameters, such as the time constants and scaling factors for LTP and LTD, are chosen to closely align with empirical findings, ensuring that the simulated synaptic dynamics reflect those observed in real biological systems. By integrating STDP into our model, we aim to capture the adaptive learning capabilities inherent to neural circuits, which is essential for accurate simulation of neural network behavior.

$$\begin{array}{cc} & \delta w=\{\begin{array}{cc} F^{+}\left(w\right)(A\cdot \text{exp}(\frac{-|\Delta t|}{\tau^{+}})-\left(A-1\right)\cdot \text{exp}(\frac{-|\Delta t|}{\tau^{x}})), & \text{if}\;\Delta t\geq 0,\\ -F^{-}(w)\;\text{exp}\;(\frac{\Delta t}{\tau^{-}}), & \text{if}\;\Delta t<0 \end{array} \end{array}$$ (11)

$$\begin{array}{cc} & F^{+}\left(w\right)=\lambda w_{0}^{1-u}w^{u}, \end{array}$$ (12)

$$\begin{array}{cc} & F^{-}\left(w\right)=\lambda \alpha w \end{array}$$ (13)

where *δw* is the synaptic strength increment, Δ*t* is the difference in firing time between the postsynaptic neuron and the presynaptic neuron, *τ* is the time constant, *w*_0_ is the unit synaptic coupling strength, *w* is the corresponding synaptic coupling strength, λ is the learning rate, *u* ∈ [0, 1], *α* is the asymmetry parameter, which measures synaptic inhibition with respect to synaptic enhancement, and *A* is the normalisation constant, and the distribution of its synaptic weights is shown in Fig 1.

### External input

The external inputs are divided into two schemes, each of which correspond to different excitatory inputs, but the inhibitory inputs remain constant.

#### Spontaneous phase

$$\begin{array}{c} I=J\cdot \sigma (t) \end{array}$$ (14)

#### Learning&recall phase

$$\begin{array}{c} I=J\cdot [\left(1-p\right)\xi^{u}+p\sigma \left(t\right)] \end{array}$$ (15)

where *σ*(*t*) is Gaussian white noise with *μ* = 0, *σ*^2^ = 1 and $\xi =\{\xi_{1}^{u},\xi_{2}^{u},\ldots ,\xi_{n}^{u}\}$ is used to denote the external Poisson input to each neuron, and the parameter in the simulation *J* = 0.8*nA*.

### Network setup

The neural network uses uniform random connections, and there are 100 neurons in the simulation, of which 80 are excitatory neurons and 20 are inhibitory neurons. (The degree of connectivity between neurons is shown in Table 3).

**Table 3:** Average connectivity.

Connection Type	Mean connectivity
E-E	0.3
E-I	0.9
I-E	0.9
I-I	0.9

### Metrics

In order to assess variations in neuronal activity over time, the most common metric is given here: the average firing rate [28–33]

$$\begin{array}{c} R\left(t\right)=\frac{1}{N}\sum_{i}^{N}r_{i}\left(t\right) \end{array}$$ (16)

where *r*_*i*_(*t*) is the firing rate of neuron *i* at time *t*, calculated using a sliding window:

$$\begin{array}{c} r_{i}\left(t\right)=\frac{1}{W}\sum_{t_{j}\in \left[t-W/2,t+W/2\right]}S_{i}(t_{j}) \end{array}$$ (17)

*W* is the sliding window size and *S*_*i*_(*t*) ∈ {0, 1} represents the firing state of neuron *i* at time *t*.

### Cell culture

Embryonic day 18 Sprague Dawley (SD) rat hippocampal neurons were isolated and cultured on microelectrode arrays (MEA) following approved procedures outlined by the Institutional Animal Care and Use Committees (IACUCs). Initially, the neurons were dissociated in DMEM buffer supplemented with 10% horse serum and 10% fetal calf serum. Subsequently, a total of 300*μ*L DMEM medium containing the dissociated neurons was evenly distributed using a transfer pipette across all the wells of an MEA (manufactured by Axion Biosystems Inc., Atlanta, GA, USA), achieving a cell density of 2.0 × 10^5^cells/cm^2^.

To facilitate optimal cell adhesion, the MEA was pre-coated with laminin and poly-d-lysine. This ensured the robust attachment of the cells to the MEA surface. The cultured neurons were maintained at a temperature of 37°C with a 5% CO_2_ environment for a duration of four weeks. During this period, the culture medium was refreshed bi-weekly by replacing half of the volume with fresh medium.

### Electrophysiological recording and stimulation

The electrophysiological recordings were conducted using the CMOS HD-MEAs system (MaxOne+, MaxWell Biosystems, Switzerland). This system is equipped with a grid of 26,400 recording electrodes, organized in a 220 × 120 layout with a 17.5*μ*m spacing, covering a 3.85 × 2.1mm^2^ area. The configuration permits simultaneous usage of up to 1024 electrodes for recording by directing them through an on-chip switch matrix to available amplifiers. To identify individual extracellular spikes, a high-pass filter with a 200Hz cutoff frequency was implemented. The detection threshold was set at 7 times the standard deviation of the average root mean square (RMS) noise level observed on each channel. Spikes occurring within a refractory period of 1ms were disregarded in the analysis.

During the 14th day in culture (DIV 14), the neural network underwent low-frequency electrical stimulation, and the ensuing patterns of activity were recorded. The electrical stimulation used the bidirectional square wave pattern, through 32 channels uniformly distributed across the entire network. The stimulation parameters were defined as having a frequency of 1Hz and an amplitude of 300 mV. After each round of stimulation was concluded, accurate timestamps of spike occurrences and the associated spike amplitudes were meticulously recorded and stored for later analysis.

### Ethics statement

This study was reviewed and approved by the Institutional Animal Care and Use Committee (IACUC) of the Chinese Academy of Medical Sciences. All animal procedures were conducted in strict accordance with the ethical standards and guidelines set forth by the institution and the American Veterinary Medical Association.

In this study, embryonic day 18 Sprague Dawley (SD) rats were used. The following measures were taken to ensure animal welfare and minimize suffering:

Euthanasia: At the conclusion of the study, the animals were humanely euthanized with an overdose of pentobarbital sodium (100 mg/kg, administered intraperitoneally), in accordance with AVMA guidelines. This method ensures a painless and stress-free process, resulting in rapid unconsciousness and death.

Animal Sacrifice: For tissue collection, animals were sacrificed by decapitation after being deeply anesthetized with isoflurane. This method was chosen to ensure a rapid and humane process, minimizing any potential distress or suffering. It is important to note the distinction between this procedure and the one described in the previous section. Pentobarbital sodium was used for euthanasia at the conclusion of the study, while decapitation was performed after anesthesia for tissue collection purposes.

Monitoring and Care: Throughout the study, animals were closely monitored for any signs of pain, distress, or discomfort. Any animal displaying signs of suffering was promptly attended to in accordance with the humane endpoints established in the protocol.

These procedures were rigorously followed to ensure the ethical treatment of the animals throughout the study, in compliance with all relevant ethical standards and institutional guidelines.

## Results

Using the above model, the simulation initially investigates the impact of Poisson stimulation [34–37], square wave stimulation [38–40], and direct current stimulation on the firing rate of neurons in terms of frequency and amplitude. Subsequently, the time taken for the network to reach steady state after introducing and removing stimulation is considered. Furthermore, the presence of memory effects in the neural network is explored by examining different stimulation schemes. Finally, experiments are conducted using real biological neural networks to compare and contrast with the conclusions drawn from the previous simulations, thereby achieving the goal of integrating experimental and simulation results for cross-validation.

### Effect of different properties of stimuli on firing rate

**Fig 2:**
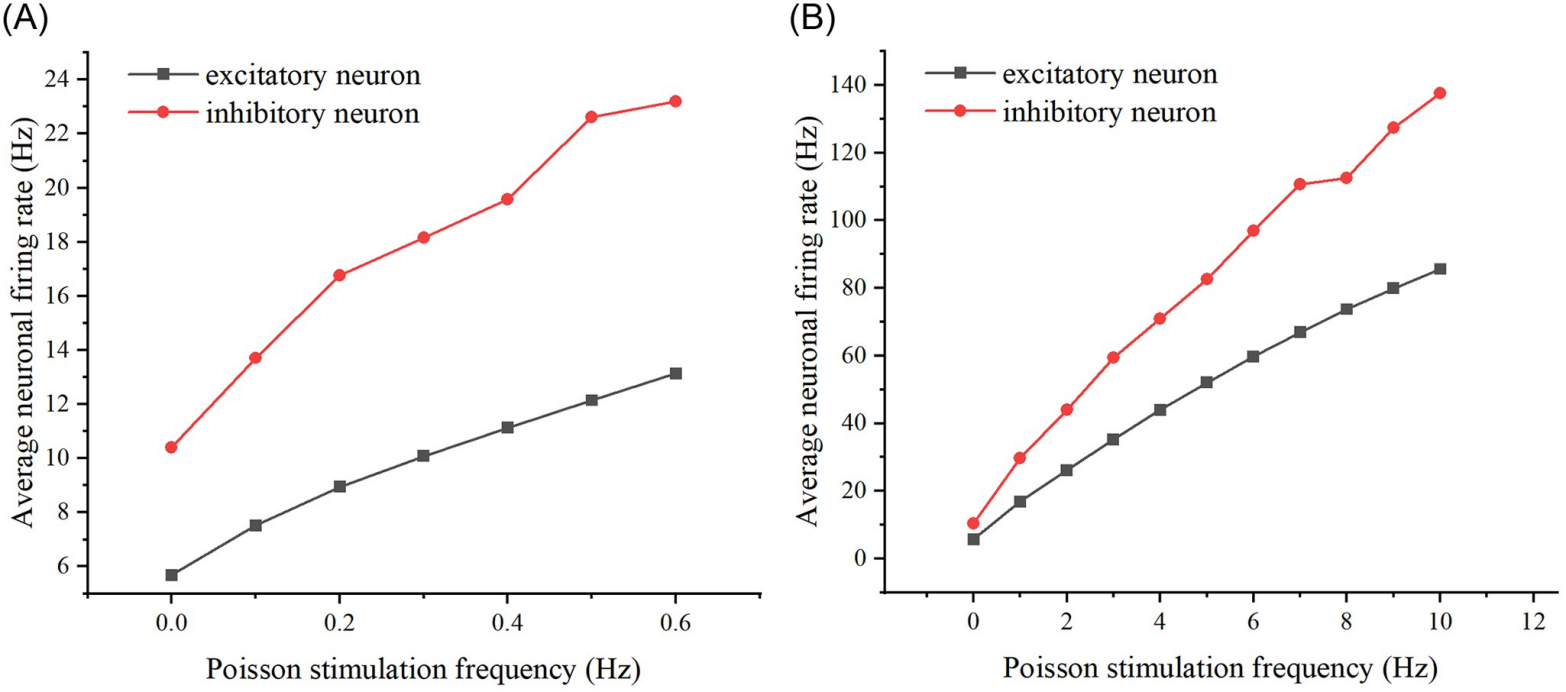
Effect of Poisson stimulation frequency on neuronal firing rate. (A) The relationship between Poisson stimulation frequency and average neuronal firing rate at low frequencies. The black curve represents excitatory neurons, while the red curve represents inhibitory neurons. (B) The relationship between Poisson stimulation frequency and average neuronal firing rate at high frequencies.

In closed-loop regulation, it is necessary to adjust the inputs to modulate the outputs for control purposes. Therefore, it is essential to understand how the neural network’s outputs change when the frequency and amplitude of the input stimulation vary. Firstly, by varying the frequency of Poisson stimulation, simulations were conducted with low and high-frequency groups. The firing rates of excitatory neurons and inhibitory neurons were recorded respectively, and the relationship graph was derived. (The relationship graph is shown in Fig 2). As can be seen by Fig 2, varying the frequency of the Poisson stimulus, whether high or low frequency, increases the average firing rate of excitatory versus inhibitory neurons as the frequency increases.

**Fig 3:**
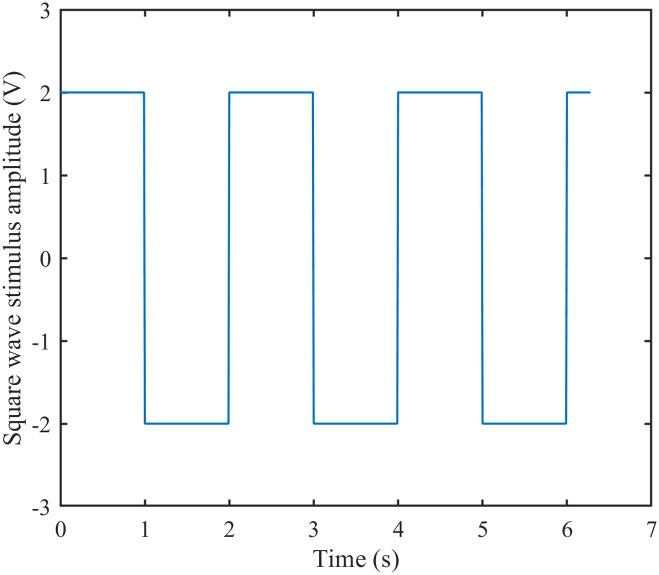
Schematic of square wave stimulation. Square wave stimulus amplitude curve over time.

**Fig 4:**
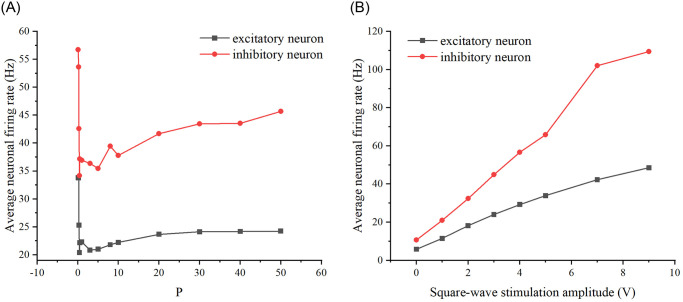
Effect of square-wave stimulus properties on neuronal firing rate. (A) Effect of square-wave stimulation frequency on firing rate. The black curve represents excitatory neurons, while the red curve represents inhibitory neurons. (B) Effect of square-wave stimulation amplitude on firing rate.

Since the square wave stimulus expression is: y = A*square(P*T), where A denotes the amplitude, P is used to regulate the period, and P*T = 2*π*, but the frequency $f=\frac{1}{T}$, so as the value of P increases, the frequency is also increasing. Similarly to Poisson stimulation, two sets of P values, small and large, are taken, and in turn the firing rates of excitatory as well as inhibitory neurons are calculated, resulting in a plot of the relationship. Additionally, since amplitude is another variable of square wave stimulation, the firing rates of neurons were similarly calculated by varying the amplitude, resulting in correlation plots. (See Fig 3 for a schematic of square wave stimulation, and Fig 4 for correlation plots depicting the relationships between frequency, amplitude, and firing rate).

As illustrated in Fig 4, when altering the frequency of the square wave stimulus, the firing rates of both excitatory and inhibitory neurons initially decrease with increasing frequency, then exhibit an increase. Similarly, modifying the amplitude of the square wave stimulus results in a consistent increase in the average firing rates of excitatory and inhibitory neurons.

**Fig 5:**
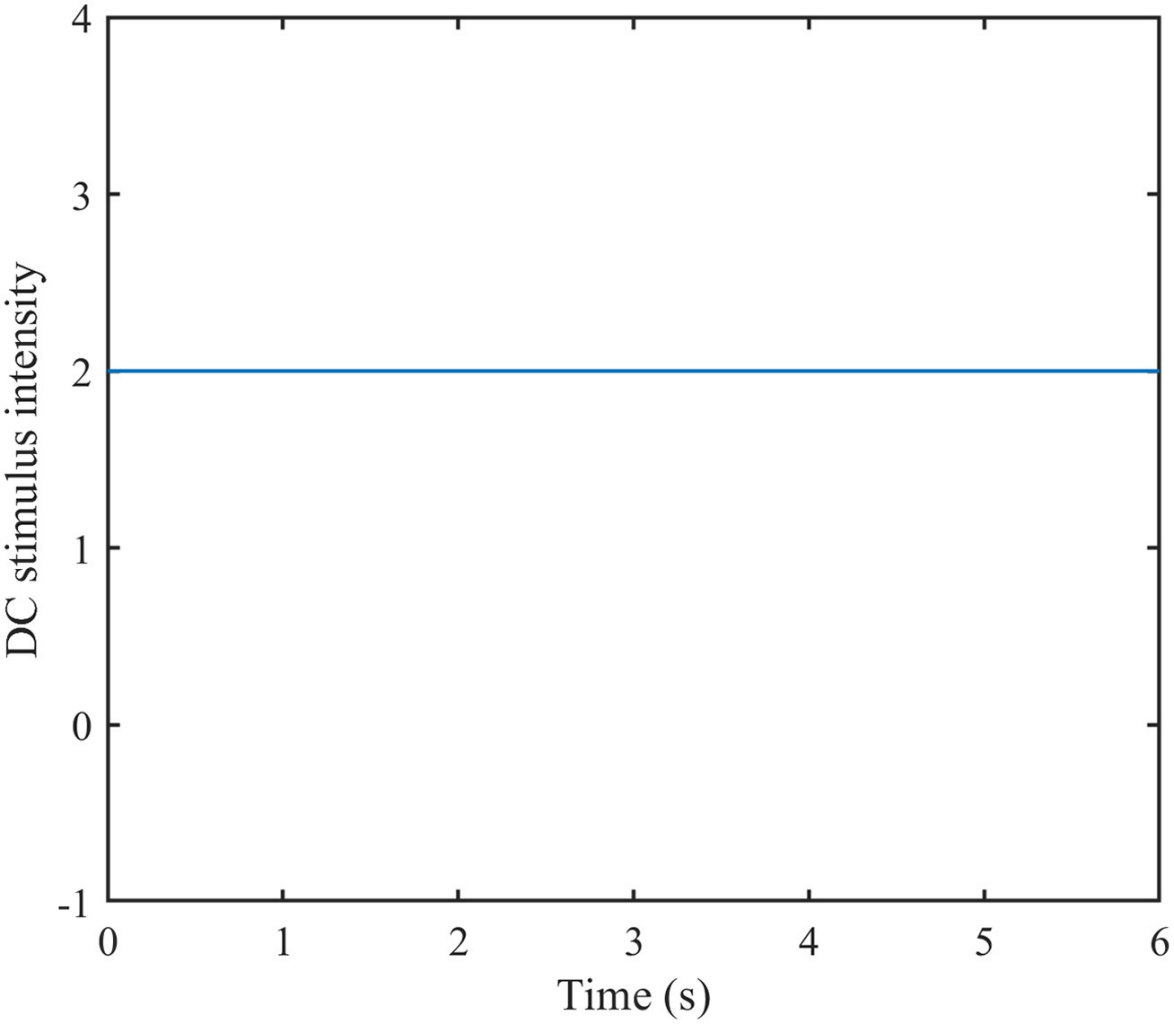
Schematic diagram of DC stimulation. DC stimulus intensity curve over time.

**Fig 6:**
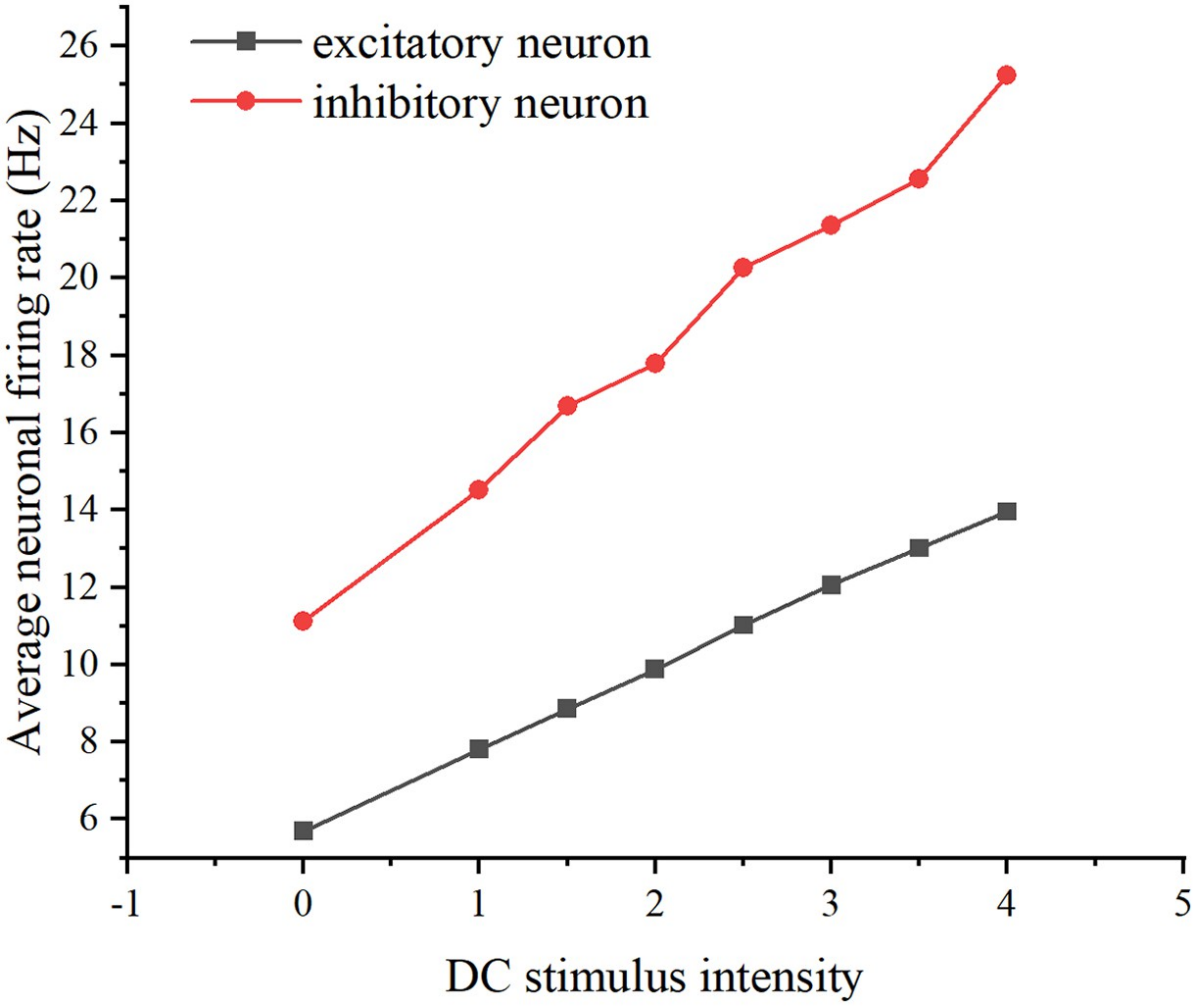
Effect of DC stimulation intensity on neuronal firing rate. The relationship between DC stimulus intensity and average neuronal firing rate. The black curve represents excitatory neurons, while the red curve represents inhibitory neurons.

Lastly, DC stimulation was introduced into the neural network (refer to Fig 5 for a schematic diagram), and the firing rates of excitatory and inhibitory neurons were individually recorded by adjusting the stimulation intensity, yielding a correlation plot. (Refer to Fig 6 for the correlation plot.).

As depicted in Fig 5, adjusting the intensity of the DC stimulus leads to an increase in the average firing rate of both excitatory and inhibitory neurons as the intensity rises.

### Stimulus stabilisation time

In closed-loop regulation, understanding the corresponding input stimuli and output responses is only possible when the network reaches a steady state. Therefore, it is crucial to discuss whether the neural network can achieve steady state, how long it takes to reach steady state after input stimulation, and how long it takes to return to steady state after cessation of stimulation. Only when the network returns to its original steady state can we explore other different input schemes.

**Fig 7:**
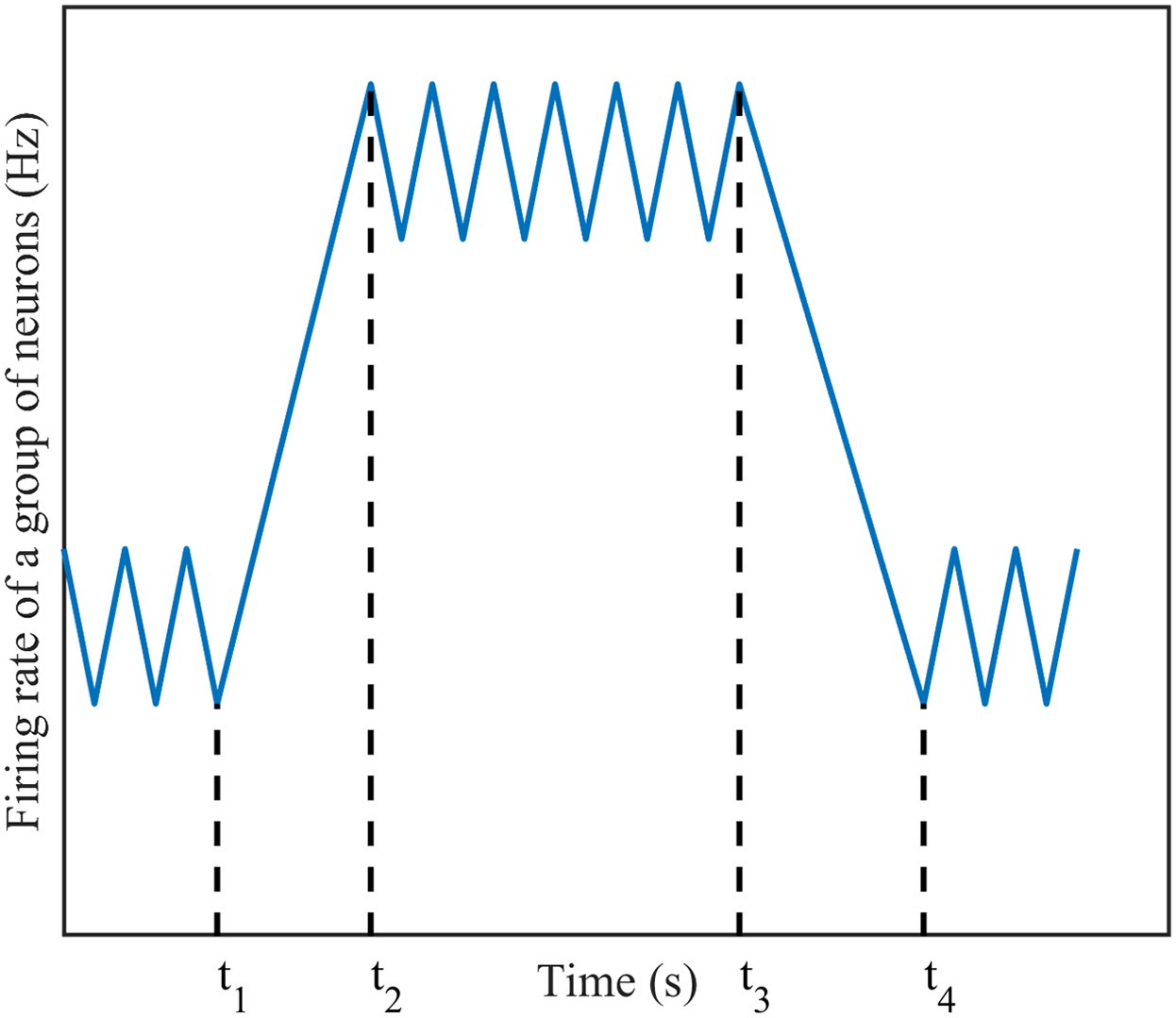
Schematic of stabilisation time. Firing rate of a group of neurons curve over time. In the figure, *t*_1_ represents the time when the stimulus is applied, *t*_2_ represents the time when the stimulus is removed, and both *t*_3_ and *t*_4_ represent the moments when the neuronal network’s firing rate stabilizes.

First, a definition of two stabilisation times is given. One is the time to reach stability after adding the stimulus *T*_1_, that is, after adding the stimulus to the neural network, the firing rate of the neural network will first show a large increase, and then return to a stable level, and the time from adding the stimulus to the firing rate to reach stability is called *T*_1_; the other is the time to reach stability after removing the stimulus *T*_2_, that is, after removing the stimulus to be added to the neural network, the neural network’s firing rate will show a significant decrease, after which it returns to a stable level, and the time from removing the stimulus to the time when the firing rate reaches stability is called *T*_2_. The stabilisation time can be seen more clearly in Fig 7, where *T*_1_ = *t*_2_ − *t*_1_ and *T*_2_ = *t*_4_ − *t*_3_.

Since stimuli are constantly added, we considered how long it takes to reach stabilisation with the addition of a stimulus and how long it takes to return stabilisation with the removal of the stimulus. We focus on DC stimulation and Poisson stimulation in this section, and compare the simulation results of the two to derive a more accurate stabilisation time for the stimulus.

**Fig 8:**
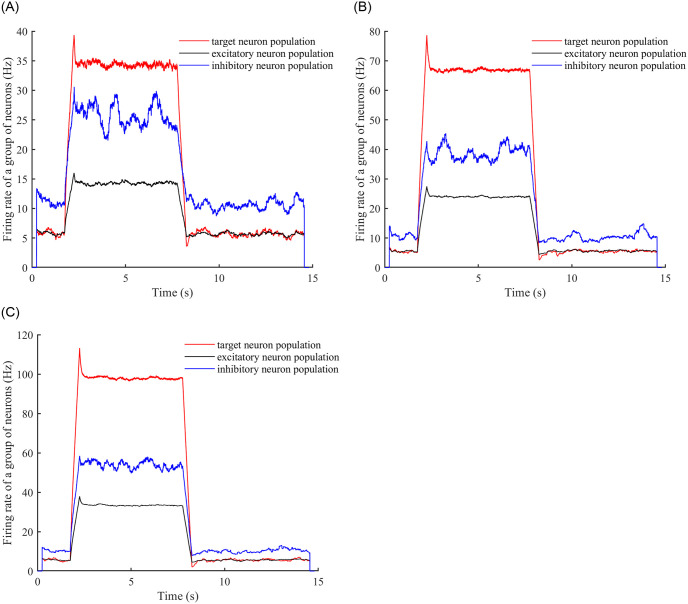
Neuron firing rates over time. (A) A DC stimulus of intensity 1 is added to the neural network, and the changes in the firing rates of the target neuron population, the inhibitory neuron population, and the excitatory neuron population are observed. (B) A DC stimulus of intensity 2 is added to the neural network, and the same changes as in (A) are observed. (C) A DC stimulus of intensity 3 is added to the neural network, and the same changes as in (A) and (B) are observed.

Considering DC stimulation first, we add DC stimulation to the neural network and observe the time needed to reach a stable firing rate and the time needed to reach stability after stabilisation and then remove the stimulation. DC stimuli of intensities 1, 2, and 3 were added, and the stimulus was added at time 2 seconds and stopped at time 8 seconds. (See Fig 8 for a graph of firing rate versus time.).

As can be seen from Fig 8, for DC stimuli of intensity 1, 2 and 3, the time to reach stability after inputting the stimulus is 0.6s, 0.66s and 0.7s respectively, and the time to reach stability after removing the stimulus is 0.72s, 0.8s and 0.82s respectively, and the values of the stable firing rate are 34.5, 67.5 and 98.2 respectively.

**Fig 9:**
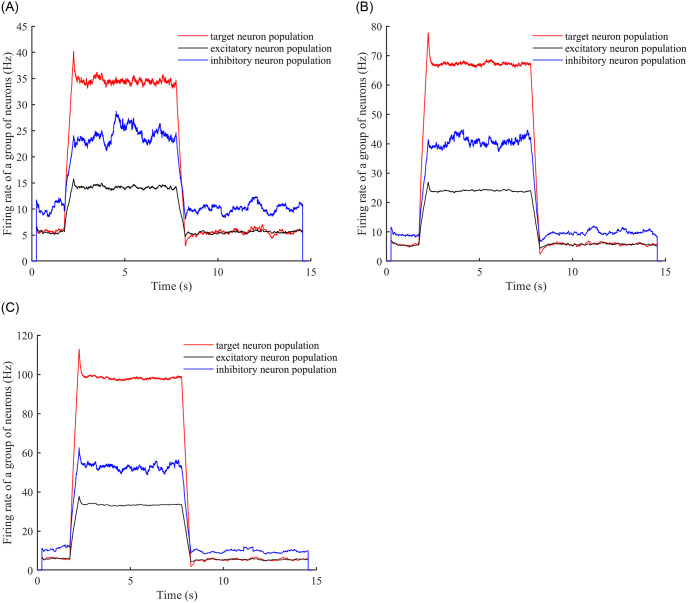
Neuron firing rates over time after changing the E-E connecting edge probability of the neural network. (A) A DC stimulus of intensity 1 is added to the neural network. (B) A DC stimulus of intensity 2 is added to the neural network. (C) A DC stimulus of intensity 3 is added to the neural network.

The above results are all based on a neuronal network with an E-E concatenation probability of 0.3, we set the E-E concatenation probability to 0.1 to do the same simulation: (see Fig 9).

As can be seen from Fig 9, the neural network with E-E concatenated edge probability of 0.1, for DC stimuli of intensity 1, 2 and 3, the time to reach stability after inputting the stimulus is 0.65s, 0.69s, 0.72s, and the time to reach stability after removing the stimulus is 0.72s, 0.83s, 0.82s, and the values of the stable firing rate are 34.7Hz, 67.3Hz, respectively, 98.3Hz.

**Fig 10:**
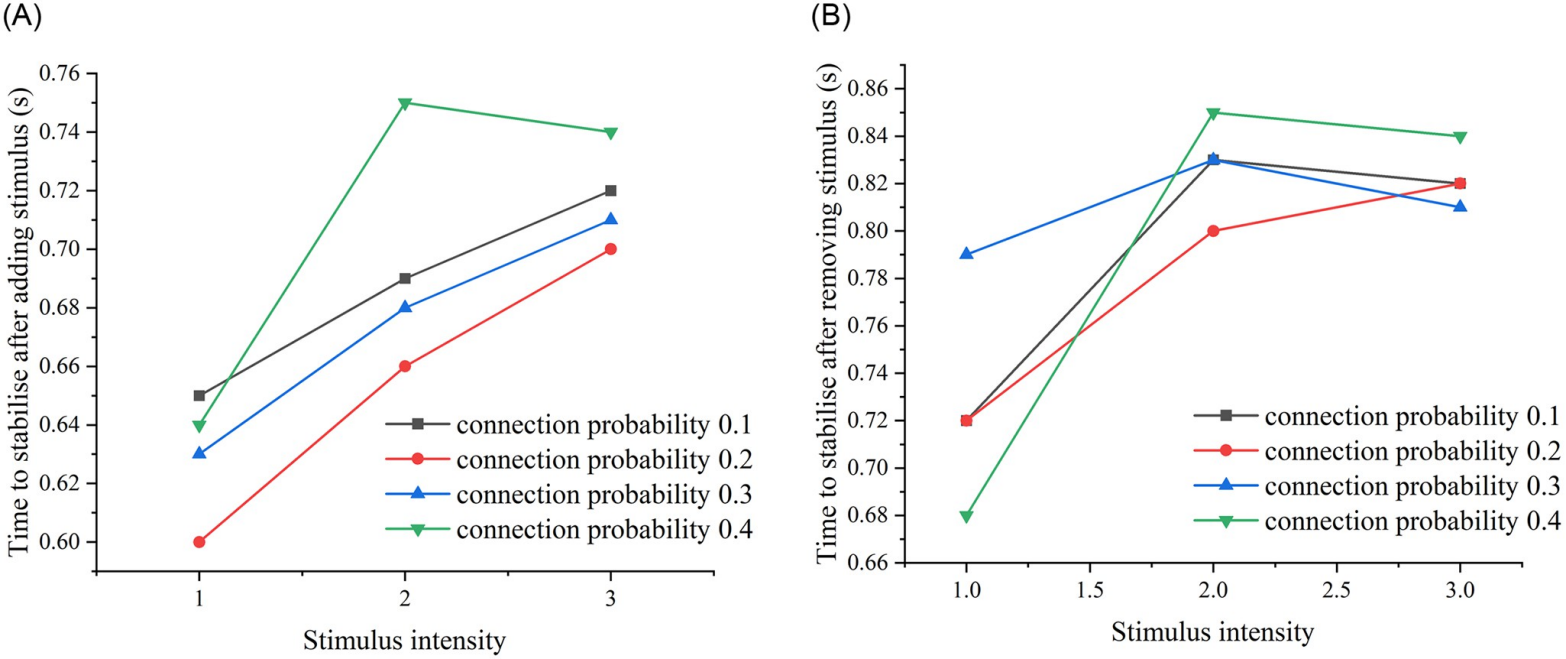
Time required to reach stabilization after adding and removing stimuli from the neural network. (A) The time required for the neural network to reach stability after adding DC stimuli of intensities 1, 2, and 3, measured across four cases with E-E concatenation probabilities of 0.1, 0.3, 0.5, and 0.7, respectively. (B) The time required for the neural network to reach stability after removing DC stimuli of intensities 1, 2, and 3, measured across the same four cases with E-E concatenation probabilities of 0.1, 0.3, 0.5, and 0.7.

In order to conclude the relationship between the stabilisation time and the edge probability, we simulated the stabilisation time of the network with E-E edge probability of 0.5 and 0.7, and compared the time to stabilise after stimulation with the stimulus intensity graphs, and the time to stabilise after removing the stimulus with the stimulus intensity graphs of the four edge probability cases: (see Fig 10).

As can be seen from Fig 10, for the four E-E concatenation probability cases, we find that the time required to reach stabilisation with the addition of the stimulus, and the time required to reach stabilisation with the removal of the stimulus do not increase with the increase of the E-E concatenation probability, but rather, the two stabilisation times reach a minimum at a concatenation probability of 0.3, and are not positively correlated.

**Fig 11:**
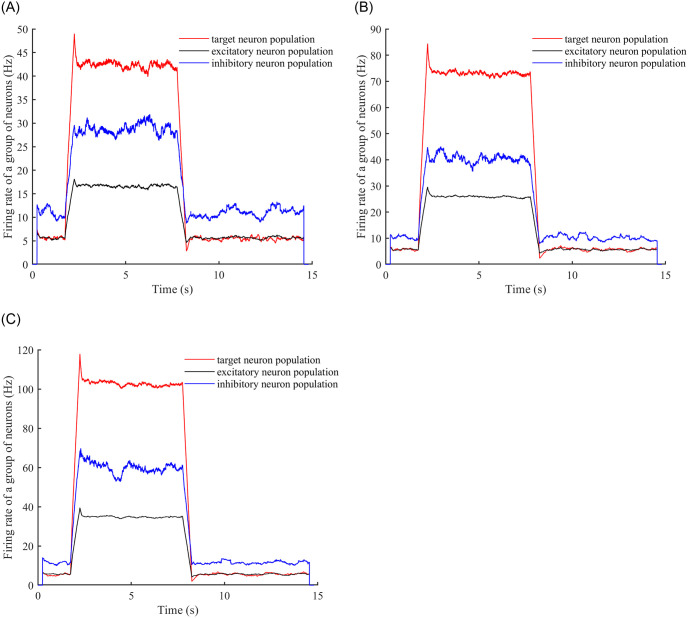
Neuron firing rates over time. (A) Observing changes in the firing rates of the target neuron population, inhibitory neuron population, and excitatory neuron population after adding a Poisson stimulus with a frequency of 1 Hz to the neural network. (B) Observing the same changes as in (A) after adding a Poisson stimulus with a frequency of 2 Hz to the neural network. (C) Observing the same changes as in (A) and (B) after adding a Poisson stimulus with a frequency of 3 Hz to the neural network.

Secondly, consider the Poisson stimulus, from the previous conclusion in the E-E contiguous edge probability of 0.3, after adding the stimulus, remove the stimulus to reach the stability of the time required for the minimum, so we only consider the E-E contiguous edge probability of 0.3 case of the neural network. We add Poisson stimuli to the neural network and observe the time required to reach a stable firing rate as well as the time required to reach stability after stabilisation and then removing the stimuli. Poisson stimuli with frequencies of 1 Hz, 2 Hz, and 3 Hz were added, and the stimulus was added at time 2 seconds and stopped at time 8 seconds. (See Fig 11 for a plot of firing rate over time).

It can be seen from Fig 11 that the neural network with E-E concatenated probability of 0.3, for Poisson stimuli with frequencies of 1 Hz, 2 Hz, and 3 Hz, reaches stability after inputting the stimulus in 0.59 s, 0.66 s, and 0.68 s, and after removing the stimulus in 0.73 s, 0.79 s, and 0.89 s, with stabilised firing rate values of 42.6Hz, 73.2Hz, 102.7Hz.

**Fig 12:**
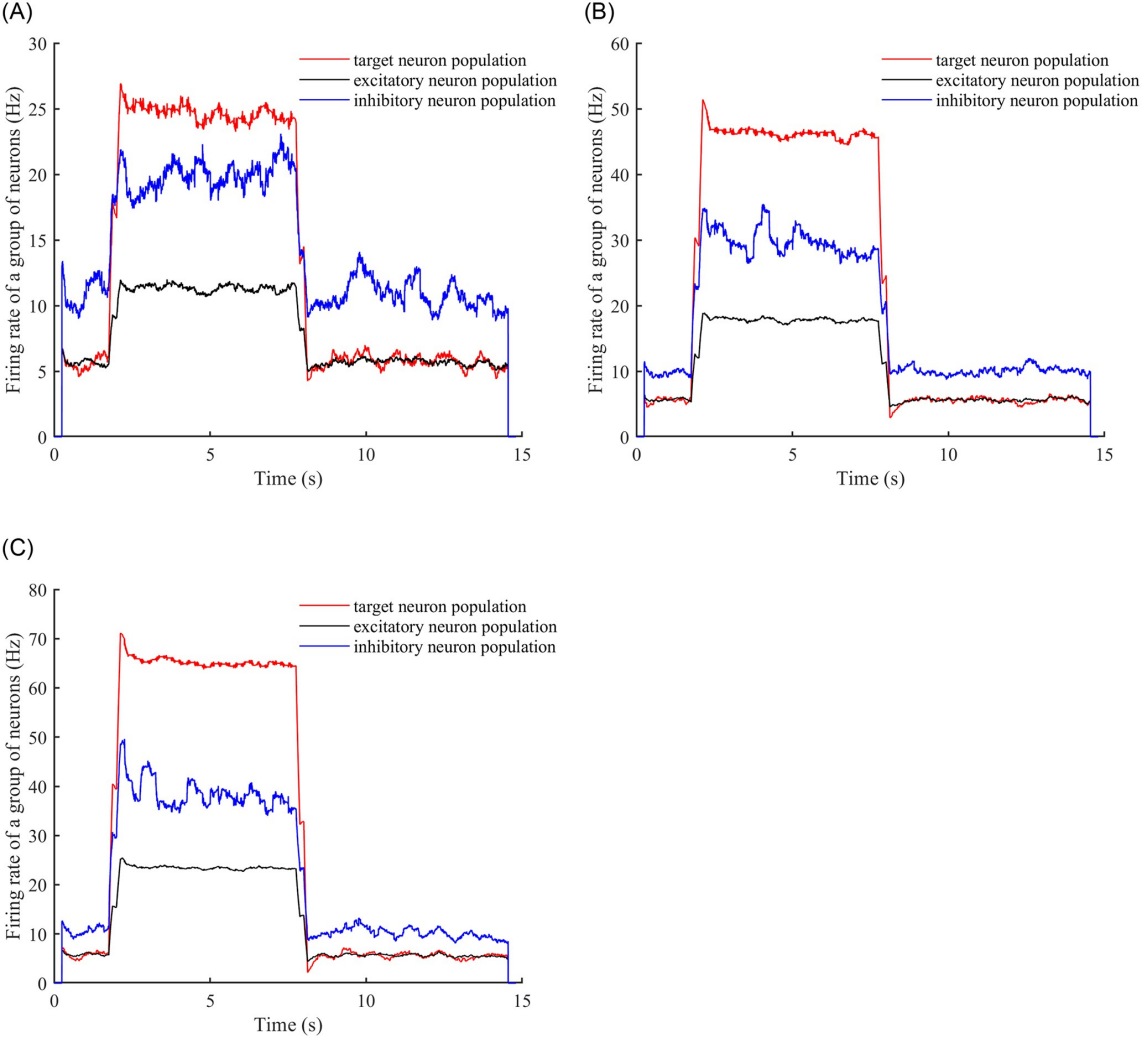
Neuron firing rates over time. (A) Observing the changes in the firing rates of the target neuron population, inhibitory neuron population, and excitatory neuron population after adding a square wave stimulus with an amplitude of 1V to the neural network. (B) Observing the same changes as in (A) after adding a square wave stimulus with an amplitude of 2V to the neural network. (C) Observing the same changes as in (A) and (B) after adding a square wave stimulus with an amplitude of 3V to the neural network.

Finally, consider the square wave stimulus, again considering only the neural network for the case where the E-E concatenation probability is 0.3. We introduce a square wave stimulus into the neural network and observe the time required to achieve a stable firing rate, along with the time taken to return to stability after stabilizing and subsequently removing the stimulus. Square wave stimuli with amplitudes of 1V, 2V, and 3V were applied at 2 seconds and discontinued at 8 seconds. (Refer to Fig 12 for a plot depicting the relationship between firing rate and time.).

Based on Fig 12, it is evident that the neural network with an E-E concatenated edge probability of 0.3 achieves stability after receiving square wave stimuli with amplitudes of 1V, 2V, and 3V. The times to reach stability upon stimulus application are 0.53s, 0.61s, and 0.64s, respectively. Similarly, the times to reach stability after the stimulus is removed are 0.54s, 0.67s, and 0.74s. The values of the stable firing rate under these conditions are 24.5 Hz, 45.8 Hz, and 65.2 Hz, respectively.

### Comparison of different stimulus schemes

In closed-loop control, we need to adjust the stimulation based on the system’s output. Therefore, it’s crucial to examine if the system exhibits memory effects from previous stimulations. If memory effects are present, fine-tuning based on the correspondence between input and output is required to design effective stimulation strategies. For instance, if memory effects lead to an increase in network firing rate, an error compensation scheme needs to be provided; otherwise, it may result in inaccurate closed-loop control.

Through the examination of the time required for the neural network to stabilize after the addition and cessation of stimuli in the preceding section, the three types of stimuli—DC stimulus, Poisson stimulus, and square wave stimulus—were compared. It was observed that the time to reach stability after adding a stimulus was approximately 0.8s, while the time to reach stability after stopping a stimulus was about 1s. Therefore, to mitigate the influence of the previous stimulus on subsequent ones, it is necessary to pause the previous stimulus for 1s before introducing the next stimulus. In this section, we investigate the presence of a memory effect in the neural network by employing various stimulus schemes.

#### Adding DC stimulation

**Fig 13:**
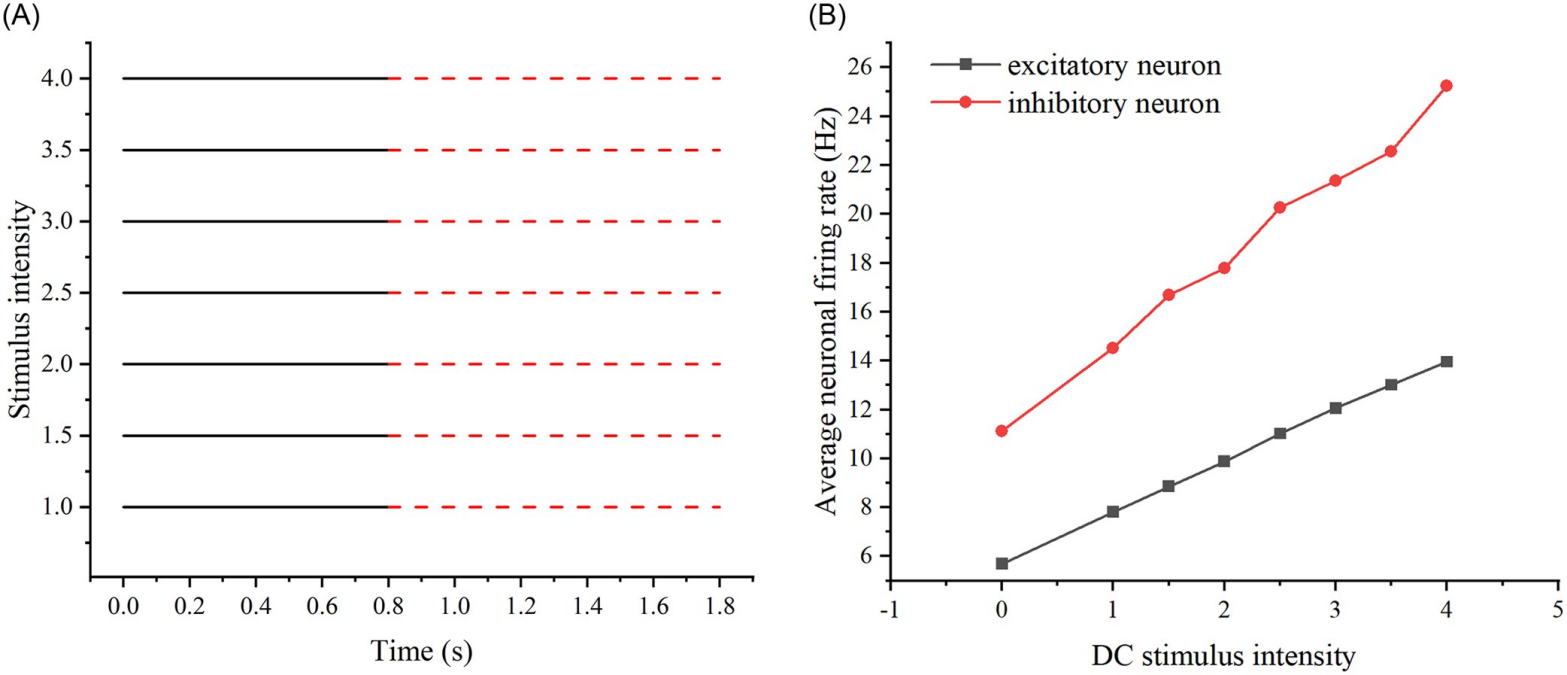
Results of adding the first stimulation scheme to the neural network. (A) Specific stimulation modality of scheme 1: A stimulus was applied to the neural network for 0.8 s and then stopped for 1 s. (B) Changes in the firing rates of excitatory and inhibitory neurons when DC stimulation, as shown in scheme 1, of increasing intensity was added to the neural network.

Firstly, we examine the DC stimulation, which is the initial stimulation scheme. Stimulation is directly applied to the network for a duration of 0.8s, and the stimulation is ceased at 1s. We observe the network’s firing rate. Several independent experiments are conducted, with each experiment being unaffected by previous ones. In each experiment, the stimulus size is incrementally increased from 1 to observe the network’s firing rate. It is ensured that multiple experiments with different stimulus strengths do not influence each other. (Refer to Fig 13 for the results of Scheme 1.).

**Fig 14:**
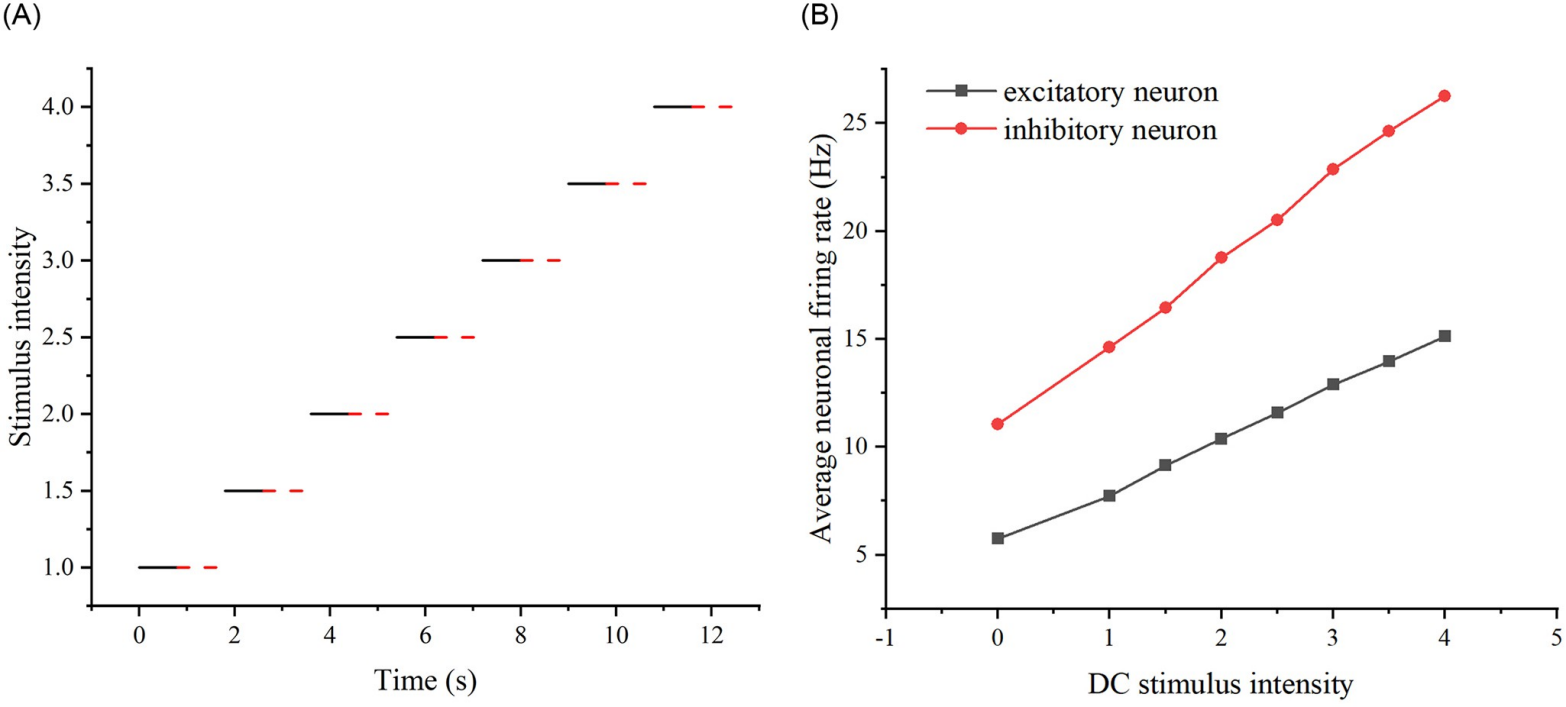
Results of adding the second stimulation scheme to the neural network. (A) Specific stimulation modality of scheme 2: Successive addition of stimulation with increasing intensity to the same neural network for 0.8 s, followed by a 1 s pause. (B) Changes in the firing rates of excitatory and inhibitory neurons when DC stimulation, as shown in scheme 2, of increasing intensity was added to the same neural network.

The second stimulus scheme involved applying a stimulus to the network with an action time of 0.8s and a cessation time of 1s. Subsequently, another stimulus was administered in the same experiment, with the stimulus size increasing in increments of 0.5. Again, the action time was 0.8s and the cessation time was 1s. This process was repeated until the stimulus size reached 4. After each stimulus was stopped, the network’s firing rate was observed. Although the different sizes of stimuli were sequentially added to the same experiment and spaced 1s apart to minimize mutual influence, there may still be an effect. (Refer to Fig 14 for the results of scheme 2.).

**Fig 15:**
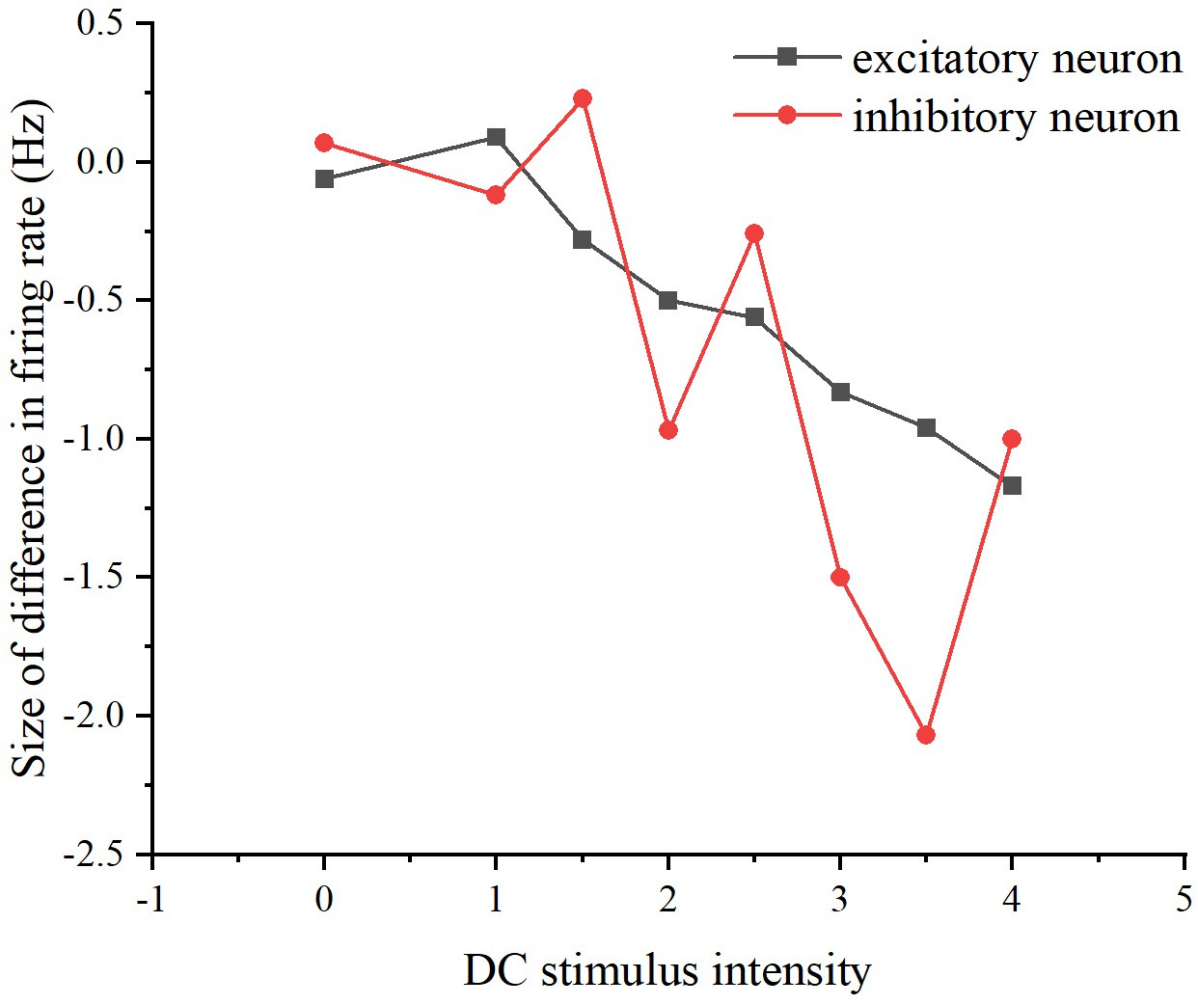
Differences in network firing rates between the two stimulation schemes. The relationship between DC stimulus intensity and the difference in firing rate. The black curve represents excitatory neurons, while the red curve represents inhibitory neurons.

Among them, Fig 13 displays multiple experiments with different intensities of stimuli. The solid line indicates the presence of stimuli, while the dashed line indicates the absence of stimuli. Fig 14 illustrates one experiment with different sizes of stimuli. Similarly, the solid line represents the presence of stimuli, and the dashed line represents the absence of stimuli. To observe whether adding stimuli of the same intensity yields the same firing rate under the two stimulation schemes, i.e., whether there is a memory effect in the neural network, we calculated the difference between the firing rates of the two schemes. Specifically, we subtracted the firing rate of scheme 1 from the firing rate of scheme 2 (see Fig 15). If the difference is negative, it indicates that the previously added stimulus in scheme 2 will influence the firing rate produced by the subsequently added stimulus.

We conducted experiments on the same neuronal network in various states and introduced DC stimuli to observe if the network could consistently produce the same firing rate, indicating the presence of a neural network memory effect. As depicted in Fig 15, noticeable differences in the network’s firing rate are evident. To ensure the generality of the results, we further investigated whether this phenomenon, i.e., the memory effect, persisted for the same neural network under different network properties. Specifically, we modified network properties such as edge probability and the number of target neurons to examine whether the discrepancy in firing rates between the two schemes, attributed to the memory effect, remained consistent. We sequentially adjusted the edge probability and the number of target neurons to repeat the stimuli in both schemes.

**Fig 16:**
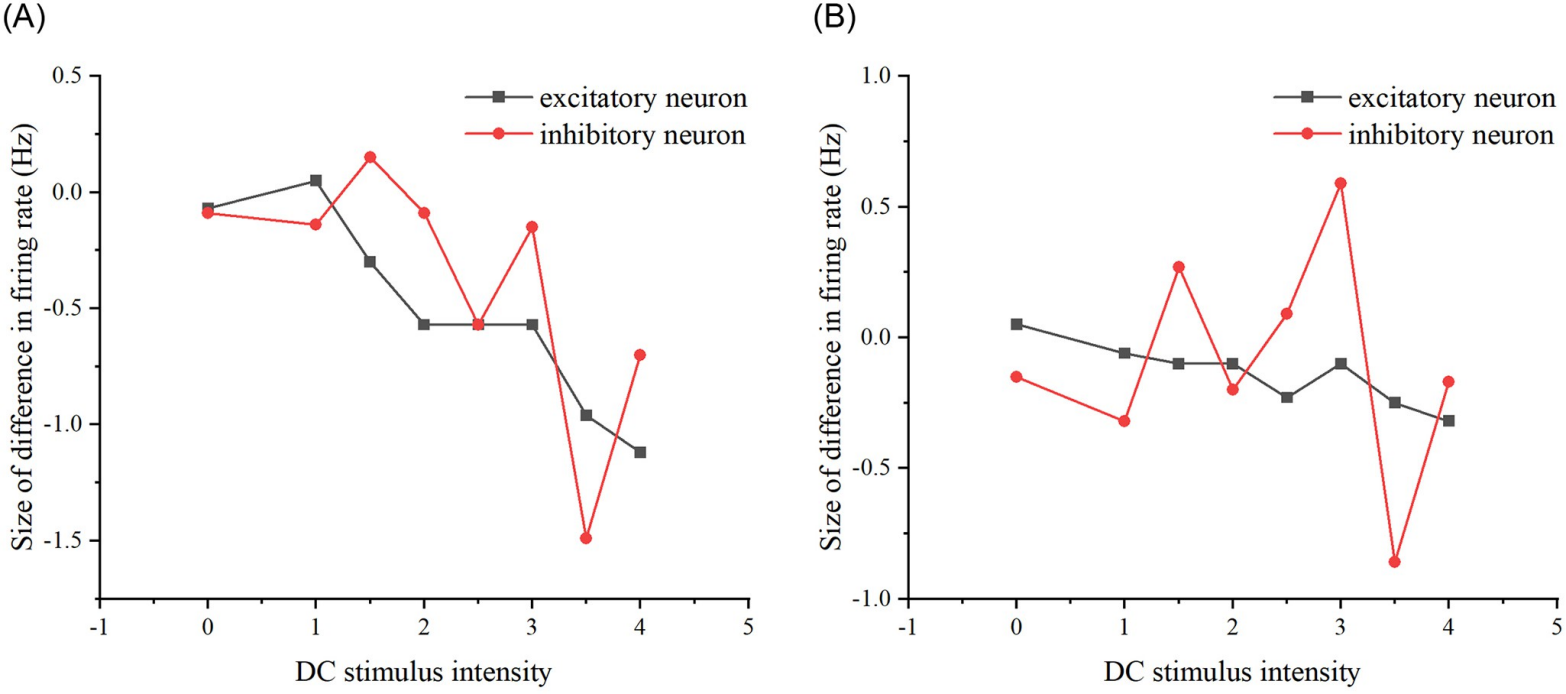
Differences in network firing rates for the two stimulation schemes that exist after modifying the properties of the neural network. (A) The difference in firing rates between the two stimulation schemes as DC stimulus intensity increases, after modifying the probability of connecting edges in the neural networks to 0.3. (B) The difference in firing rates between the two stimulation schemes as DC stimulus intensity increases, after modifying the number of target neurons in the neural network to 8.

First, the connecting edge probability was modified, the original neural network was E-E: 0.3; E-I: 0.9; I-E: 0.9; I-I: 0.9, and the modified neural network was E-E: 0.3; E-I: 0.3; I-E: 0.3; I-I: 0.3. Second, the number of target neurons was modified, the original neural network was 24, and the modified neural network was 8. (see Fig 16).

From Figs 15 and 16, it can be seen that whether modifying the probability of connecting edges or the number of target neurons, there is a certain difference in the firing rate of the neural network under the two schemes, so there is an effect of the previous stimulus on the neural network, i.e., there is a memory effect on the neural network, of which the memory effect of the original neural network is the most obvious in the stimulus intensity of the size of 3-4, and the memory effect of the neural network after modifying the probability of connecting edges is the most obvious in the stimulus intensity The original neural network has the most obvious memory effect when the stimulus intensity is 3-4, the neural network after modifying the probability of connecting edges has the most obvious memory effect when the stimulus intensity is 3.5 and the neural network after modifying the number of target neurons has the most obvious memory effect when the stimulus intensity is 3.5. Therefore, overall, the most obvious memory effect exists when the added current strength is 3.5 and its neighborhood.

#### Adding Poisson stimulation

**Fig 17:**
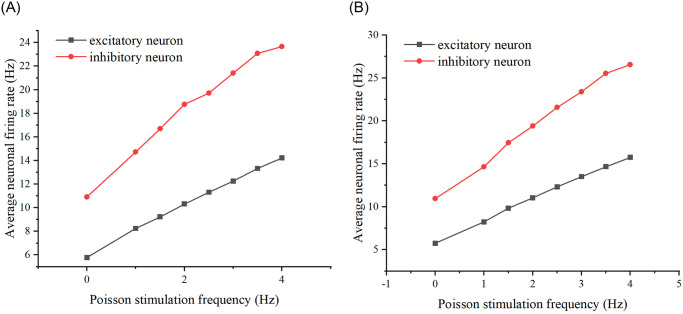
Two schemes of Poisson stimulation were added to the neural network. (A) The variation in firing rates of excitatory and inhibitory neurons after adding Poisson stimuli from scheme 1 to the neural network. (B) The variation in firing rates of excitatory and inhibitory neurons after adding Poisson stimuli from scheme 2 to the neural network.

Secondly, consider Poisson stimulation, the two stimulation schemes are the same as DC stimulation, the only difference is that the DC stimulation intensity size is replaced with the Poisson stimulation frequency size, and the Poisson stimulation of Scheme 1 and Scheme 2 is applied to the original neural network, and observe the relationship between the neural network firing rate and the stimulation frequency. (See Fig 17).

**Fig 18:**
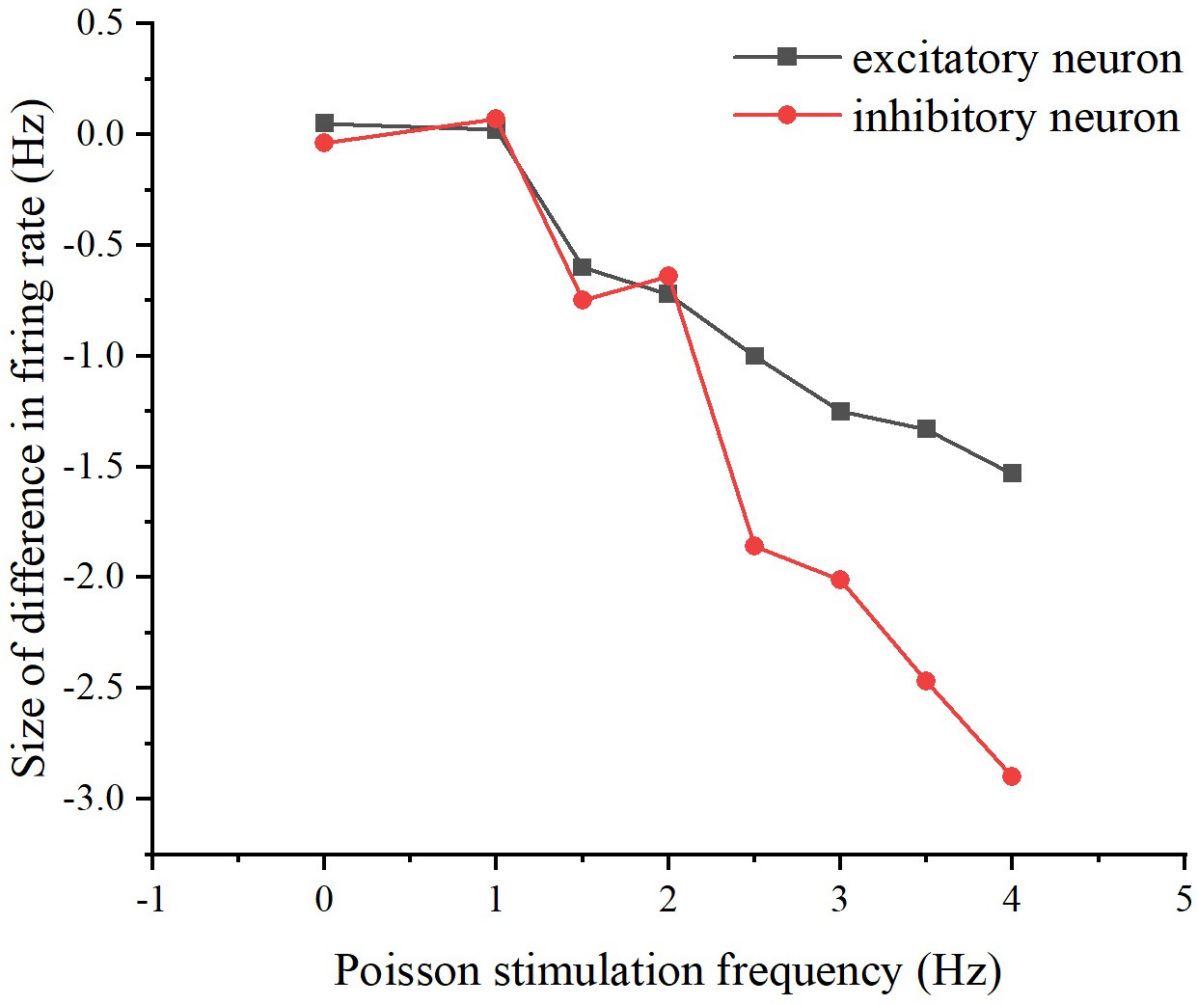
Differences in network firing rates between the two stimulation schemes. The relationship between Poisson stimulation frequency and the difference in firing rate. The black curve represents excitatory neurons, while the red curve represents inhibitory neurons.

**Fig 19:**
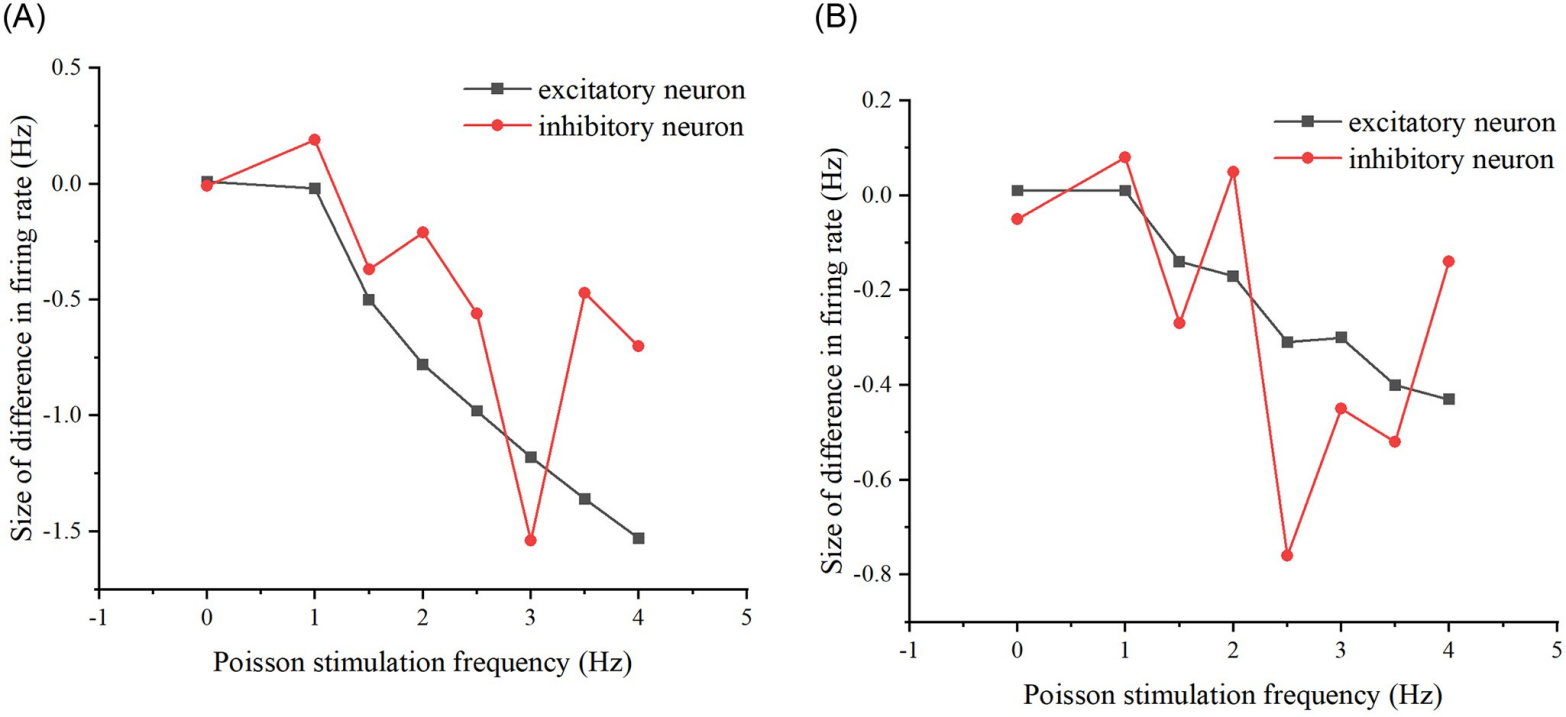
Difference in the network firing rate of the two stimulation schemes present after modifying the properties of the neural network. (A) The difference in firing rates between the two stimulation schemes as Poisson stimulation frequency increases, after modifying the probability of connecting edges in the neural networks to 0.3. (B) The difference in firing rates between the two stimulation schemes as Poisson stimulation frequency increases, after modifying the number of target neurons in the neural network to 8.

Subsequently, the firing rates of excitatory and inhibitory neurons differed between the two schemes to observe the neural network’s memory for the Poisson stimulus (see Fig 18). The stimulus was repeated for the two schemes by similarly modifying the concatenation probability and the number of target neurons sequentially, as in the previous section (see Fig 19).

From Figs 18 and 19, it can be seen that whether modifying the probability of connecting edges or the number of target neurons, there is a certain difference in the firing rate of the neural network under the two schemes, so there is an effect of the previous stimulation on the neural network, i.e., there is a memory effect on the neural network, of which the memory effect of the original neural network is the most obvious when the stimulation frequency size is 2.5-4Hz, the memory effect of the modified probability of connecting edges is the most obvious when the The original neural network has the most obvious memory effect when the stimulus frequency is 2.5-4Hz, the neural network after modifying the probability of connecting edges has the most obvious memory effect when the stimulus frequency is 3Hz, and the neural network after modifying the number of target neurons has the most obvious memory effect when the stimulus frequency is 2.5-3.5Hz. So overall, the memory effect present is most pronounced when the frequency of the added stimulus is 3Hz and its neighborhood.

#### Adding square wave stimulation

**Fig 20:**
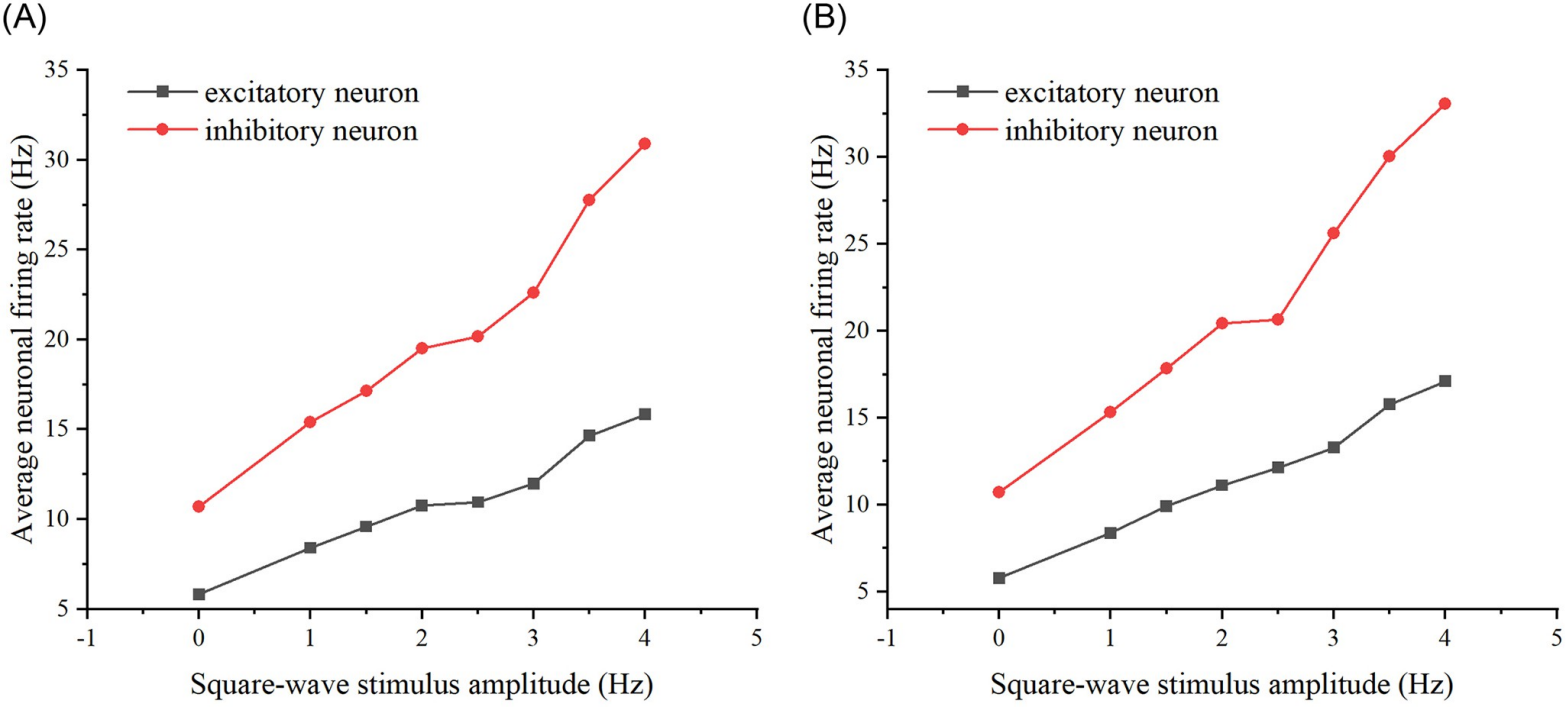
Two schemes of square wave stimulation were added to the neural network. (A) The variation in firing rates of excitatory and inhibitory neurons after adding square wave stimuli from scheme 1 to the neural network. (B) The variation in firing rates of excitatory and inhibitory neurons after adding square wave stimuli from scheme 2 to the neural network.

Finally, square wave stimulation is introduced, with the two stimulation schemes being the same as before. The distinction lies in replacing the variable with the size of the square wave stimulation amplitude. Both scheme 1 and scheme 2 square wave stimulations are applied to the original neural network, and the relationship between the neural network’s firing rate and the stimulation amplitude is observed (See Fig 20).

**Fig 21:**
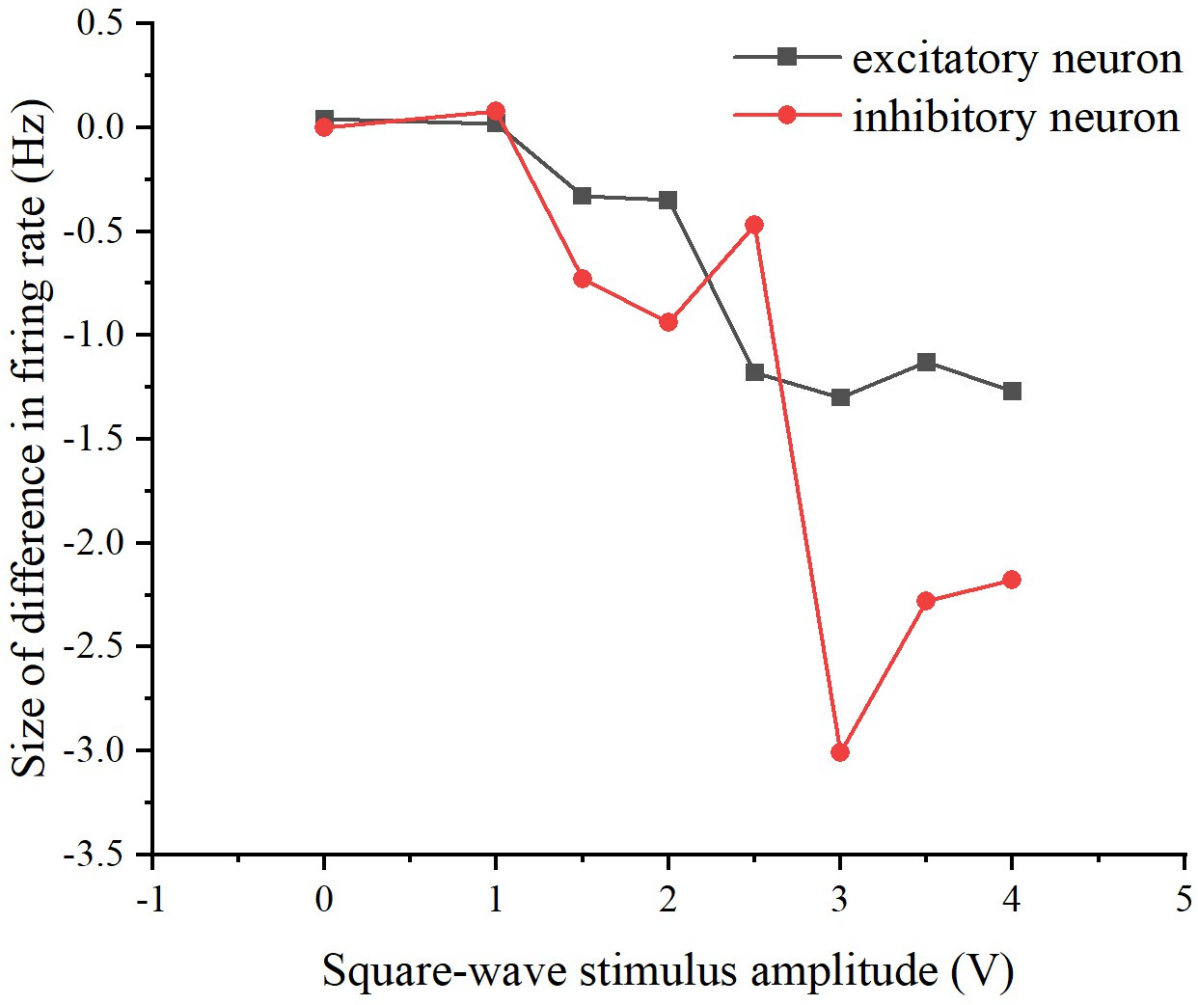
Differences in network firing rates between the two stimulation schemes. The relationship between square-wave stimulus amplitude and the difference in firing rate. The black curve represents excitatory neurons, while the red curve represents inhibitory neurons.

**Fig 22:**
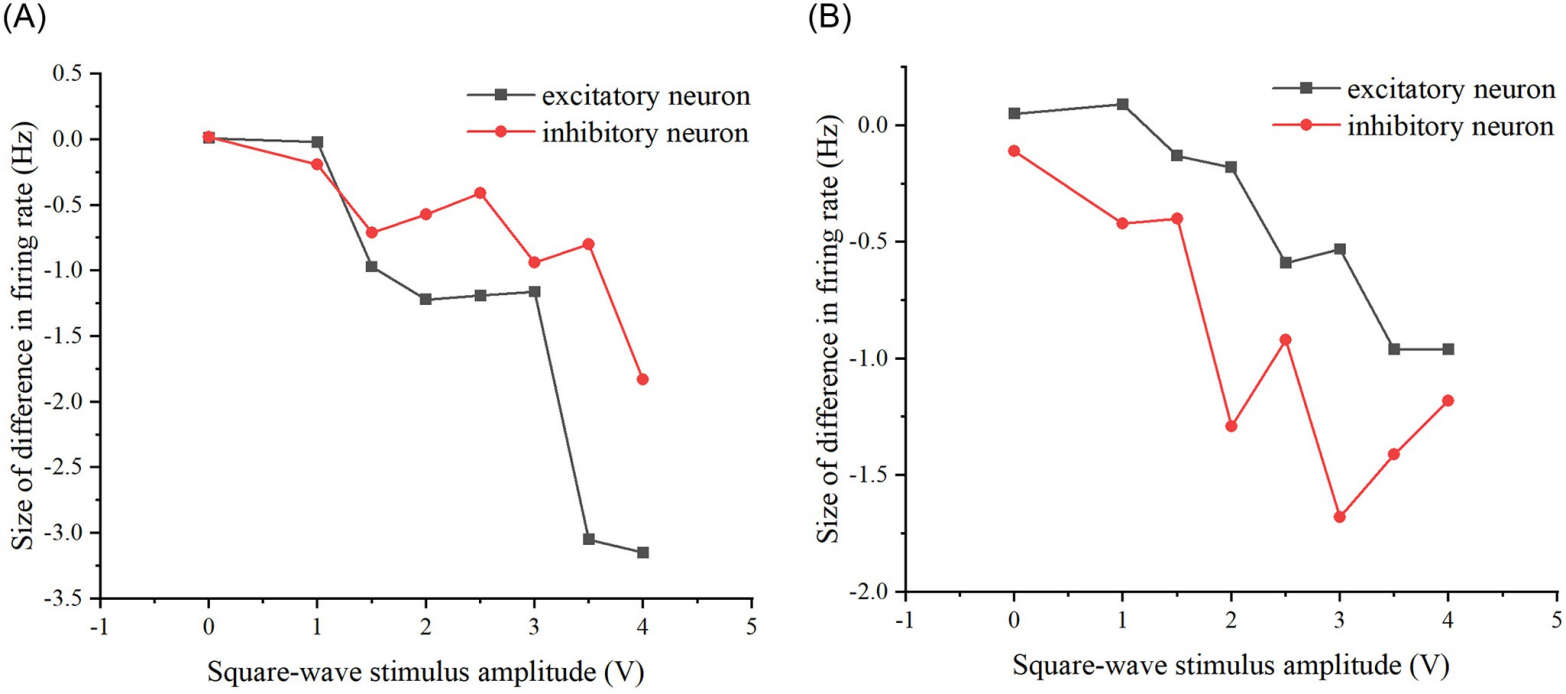
Difference in the network firing rate of the two stimulation schemes present after modifying the properties of the neural network. (A) The difference in firing rates between the two stimulation schemes as the amplitude of square wave stimulation increases, after modifying the probability of connecting edges in the neural networks to 0.3. (B) The difference in firing rates between the two stimulation schemes as the amplitude of square wave stimulation increases, after modifying the number of target neurons in the neural network to 8.

The firing rates of excitatory and inhibitory neurons differed between the two schemes to observe the memory of the neural network for the square-wave stimulus (see Fig 21). The stimuli for the two schemes were similarly repeated by modifying the concatenation probability and the number of target neurons sequentially, as in the previous two subsections (see Fig 22).

From Figs 21 and 22, it is evident that whether modifying the probability of connecting edges or the number of target neurons, there is a discernible difference in the firing rate of the neural network under the two schemes. This suggests an influence of the preceding stimulus on the neural network, indicating a memory effect. Specifically, the memory effect of the original neural network is most pronounced when the stimulus amplitude is 3V. Similarly, the neural network after modifying the probability of connecting edges exhibits the most noticeable memory effect when the stimulus amplitude size is 3V, and the neural network after modifying the number of target neurons demonstrates the most apparent memory effect when the stimulus amplitude size is 3V. Overall, the memory effect is most pronounced when the amplitude of the added stimulus is 3V and its vicinity.

Combining the effects of the various properties of the three different stimuli on the memory effect in the preceding three subsections, it can be inferred that regardless of whether it is DC stimulation, Poisson stimulation, or square-wave stimulation, the neural network will exhibit memetic properties in response to them. Particularly under conditions of high-intensity DC stimulation, high-frequency Poisson stimulation, and high-amplitude square-wave stimulation, the memory effect is most pronounced.

### Experimental verification

The previous section primarily utilized model simulations to explore the relationship between input and output of neural networks and to investigate the existence of a memory effect. In contrast, this section primarily conducts experiments on real biological neural networks. The objective is to integrate model simulations with real experiments, enhancing the persuasiveness of the conclusions and providing researchers with more effective guidance for exploring stimulus modulation in future studies.

#### Experimental ideas

Using a neural network consisting of a large number of cultured real neurons, which included more than 1000 electrodes. Firstly, multi-electrode stimulation was performed on selected areas with higher firing rates, employing the stimulation modes outlined in the respective schemes; secondly, the average firing rate of the network was recorded, and lastly, the experimental data were analysed comparatively and conclusions were drawn.

Among them, scheme 1 is independent stimulation, with a long interval between adjacent stimuli, and two adjacent stimuli can be regarded as independent stimuli so that the neural network has enough time to recover to the original state; scheme 2 is continuous stimulation, with a short interval between adjacent stimuli, and thus the neural network is unable to recover to the original state. The comparison of experimental data is divided into within-scheme and between-scheme, within-scheme comparison to verify whether the stimulation parameter has a facilitating effect on the firing rate of the neural network; between-scheme comparison to compare the experimental data derived from the two stimulation schemes, to explore the memory and learning ability of the neural network.

#### Experimental step

Firstly, 10 electrodes were randomly and uniformly selected in the more prominent areas of firing in the cultured neural network, and the firing areas selected for both schemes were consistent with the electrodes. Secondly, independent stimulation was performed, in which the type of stimulation was bi-directional square wave stimulation, the stimulation frequency was 1Hz, the stimulation time was 60 seconds, and the stimulation amplitude increased from 100mV to 600mV in 100 steps, and the firing of the neural network was recorded for 2 minutes at the end of each stimulation, with a 30-minute rest period between the two neighbouring stimulations in order to allow the neural network to have enough time to recover to the original state.

Subsequently, the next day, the neural network was subjected to continuous stimulation with the same type, frequency, duration, and amplitude as in the independent stimulation, and the firing of the neural network was recorded for 2 minutes at the end of each stimulation, and the next stimulation was applied immediately after the recording was completed. Finally, the recorded experimental data were processed to calculate the average firing rate of the network, while the data obtained from the two protocols were subjected to a difference-making process to complete the plotting.

#### Experimental conclusion

**Fig 23:**
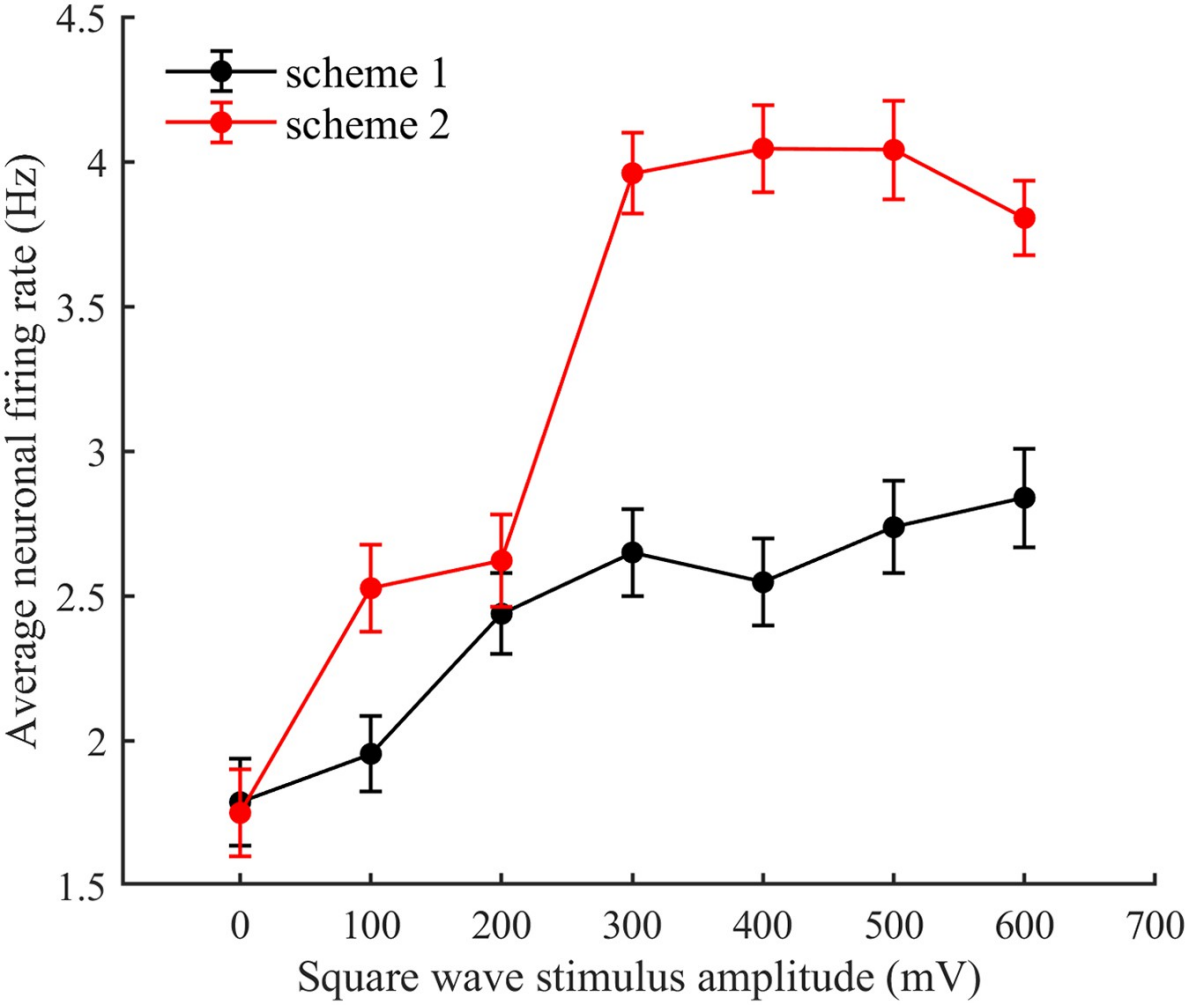
Addition of square wave stimuli for both schemes. The relationship between square wave stimulus amplitude and average neuronal firing rate. The black curve represents scheme 1, while the red curve represents scheme 2. The statistics are averaged from n = 4 wels in experiment group. Data are expressed as the mean ± SEM.

The relationship between the neural network firing rate and the stimulus amplitude was derived from the within-programme comparisons. (See Fig 23.).

It can be seen from Fig 23 that the firing rate of the neural network increases as the stimulus amplitude increases in both schemes 1 and 2. Comparing this with Figs 2, 4 and 6, this finding is found to be the same as that derived from the model simulation, thus further validating the existence of this conclusion.

**Fig 24:**
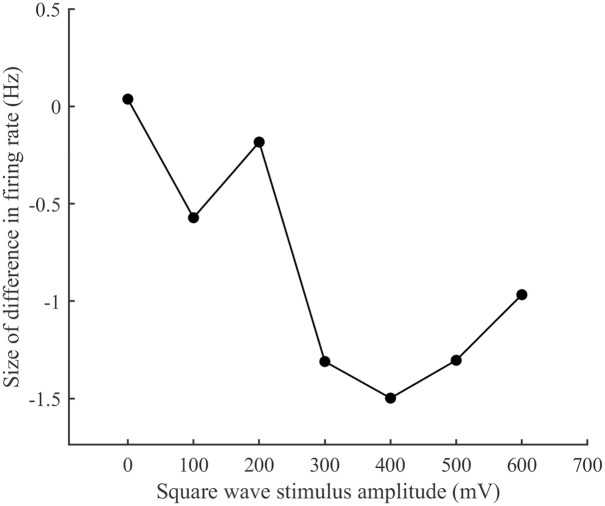
Differences in network firing rates between the two stimulation schemes. The relationship between square-wave stimulus amplitude and the difference in firing rate.

By comparing between the schemes, the neural network firing rates derived from the two schemes are made to differ, and the relationship between the size of the difference in firing rate and the stimulus amplitude is derived, so as to observe the memory of the neural network firing rate for the biphasic square wave stimulus. (See Fig 24.).

As shown in Fig 24, the neural network exhibits memory for bi-directional square wave stimuli, particularly when the stimulus amplitude is between 300-500 mV, where the memory effect is most pronounced. Model simulations indicate that the network also retains memory for both DC stimuli and Poisson stimuli, with the memory effect being especially strong for high-intensity DC stimuli or high-frequency Poisson stimuli. A comparison of these simulations with Figs 15, 18 and 21 reveals a similar trend. When combining real experimental findings with model simulations, a high degree of similarity is observed. This leads to the conclusion that, under continuous stimulation with increasing parameters, the neural network develops a memory effect for the stimulus, which is most significant when the stimulus parameters are larger.

## Discussion

Sakai et al. and Ryan Weiss et al. drew inspiration from the understanding that STDP facilitates synaptic competition and activity regulation, which in turn moderately modulates synaptic homeostasis to regulate postsynaptic activity. STDP, a widely-used online learning method, determines the update of synaptic weights based on the relative timing of presynaptic and postsynaptic peak times. Hence, they employed the STDP synaptic plasticity model and explored the average neuronal firing rate as a measure [41, 42]. Given that Poisson stimuli, square wave stimuli, and DC stimuli find broad applications in scientific research, especially in neuroscience, biology, and engineering, they serve to simulate and study the responses and dynamic behaviors of various systems [34, 37, 38, 43, 44]. Therefore, initially, concerning Poisson stimuli,square wave stimuli and DC stimuli, it was observed that the firing rate of neurons increases with the frequency, amplitude, and intensity of the stimuli. This phenomenon can be elucidated as a response enhancement phenomenon, wherein the strength of connections between synapses alters with increasing stimulus frequency. Additionally, the neuron may accumulate more potential changes in a short period, augmenting the likelihood of reaching the threshold and initiating an action potential. In essence, the interplay between synaptic plasticity and the internal mechanisms of the neuron contributes to the increased firing rate.

Secondly, to investigate the time required for the neural network to stabilize after the addition and removal of stimuli, Poisson, DC, and square wave stimuli were introduced to cross-validate each other. These three stimuli represent different types of neural stimulation. Firstly, Poisson stimulation consists of a sequence of random events, where the time interval between each stimulus pulse follows a Poisson distribution, exhibiting no fixed pattern. Secondly, DC stimulation involves a constant current stimulus, where the direction and magnitude of the current remain constant. Lastly, square-wave stimulation is characterized by an electrical stimulus with a fixed amplitude and period, presenting a square wave-shaped waveform. Each of these stimuli possesses distinct characteristics, warranting a comparison. After conducting several experiments to determine the average values, it was concluded that the stabilization time after adding the stimulus is 0.8s, while the stabilization time after removing the stimulus is 1s. These findings serve as the foundation for subsequent experimental investigations.

Finally, an investigation was conducted to determine if there exists a memory effect in the neural network under different conditions. Two distinct stimulation schemes were formulated based on the previously determined stabilization times for adding and removing stimuli. By applying these schemes and introducing DC, Poisson, and square wave stimulation, and subsequently averaging the results from multiple experiments, it was established that a memory effect indeed exists in the neural network. To assess the generalizability of these findings, modifications were made to the network properties, including the probability of connecting edges and the number of target neurons. Additionally, real biological neural networks were utilized to verify these results, confirming the presence of a memory effect when biphasic square wave stimuli were applied. In conclusion, continuous stimulation of the neural network, particularly with increasing stimulation parameters, leads to the emergence of a memory effect. This effect is most pronounced when the stimulation parameters are large. By integrating model simulations with biological experiments, the conclusion is bolstered, enhancing its credibility.

When DC stimulation was added, the memory effect was most pronounced when the current stimulation intensity was around 3.5 units. For Poisson stimulation, the memory effect was most evident when the stimulation frequency was approximately 3Hz. Similarly, with square wave stimulation, the memory effect peaked when the stimulation amplitude reached around 3V. Comparing the locations of the most prominent memory effects under different stimulus attributes revealed interesting insights. For DC and square wave stimulation, variations in neural network attributes had minimal impact on the location of the memory effect peak. However, for Poisson stimulation, modifying the neural network attributes did influence the location of the memory effect peak. Specifically, as network attributes changed, the peak memory effect gradually shifted to the left. The distinct responses induced by the three types of stimuli are primarily attributed to the randomness and constancy of the stimuli, as well as the influence of corresponding synaptic plasticity mechanisms and neuronal activation thresholds. These factors collectively shape the response patterns and dynamic properties of the neural network to various types of stimuli. The presence of memory effects in neural networks has been corroborated and partially applied in previous studies [45, 46]. Driven by the interaction between synaptic plasticity and neuronal activity patterns, memory effects enable neural networks to store and retrieve information following stimulus reception. This phenomenon underscores the foundation of neural networks and remains a focal point of research in neuroscience and cognitive science.

## Conclusion

In this paper, by constructing a neural network model as well as a neural network composed of real neurons cultured in vitro, we explored from both simulation and experimental perspectives. The final conclusion is that for various types of stimulus, the firing rate of the neural network increases with the parameters of the stimulus. Additionally, the neural network exhibits a memory effect for a wide range of stimulus types, with the memory effect being more pronounced when the stimulus parameters is higher. This provides a good pioneering theoretical study of the use of closed-loop control to achieve complex functions in the field of engineering, which is conducive to more efficient use of closed-loop control to achieve the target function by the researchers in engineering, which in turn is conducive to more in-depth investigation.

However, this paper does not explore all stimulus types in real, which is a problem to be considered in the follow-up. More types of stimuli will be considered in subsequent simulations and experiments, so as to provide a stronger support for the results of this paper, and also offer a good reference for the stimulus modulation.

## Code availability

The code for this project can be found at the following github repository: https://github.com/333sen/333sen.git.
